# Cohort-specific determinants of donor strain engraftment following multi-donor faecal microbiota transplantation in two randomised clinical trials

**DOI:** 10.1080/19490976.2025.2597628

**Published:** 2025-12-11

**Authors:** Anna H. Behling, Theo Portlock, Daniel Ho, Brooke C. Wilson, Sudarshan Paramsothy, Michael A. Kamm, Wayne S. Cutfield, Nadeem O. Kaakoush, Justin M. O’Sullivan, Tommi Vatanen

**Affiliations:** a Liggins Institute, University of Auckland, Auckland, New Zealand; b Concord Clinical School, University of Sydney, Sydney, NSW , Australia; c Department of Gastroenterology, Concord Repatriation General Hospital, Sydney, NSW , Australia; d Department of Gastroenterology, St Vincent's Hospital, Melbourne, Vic ​​​​​​, Australia; e School of Biomedical Sciences, University of New South Wales, Sydney, NSW , Australia; f MRC Lifecourse Epidemiology Unit, University of Southampton, Southampton, United Kingdom; g The Maurice Wilkins Centre, The University of Auckland, Auckland, Private Bag, New Zealand; h Institute of Biotechnology, Helsinki Institute of Life Science, University of Helsinki, Helsinki, Finland; i Department of Microbiology, Faculty of Agriculture and Forestry, University of Helsinki, Helsinki, Finland; j Research Program for Clinical and Molecular Metabolism, Faculty of Medicine, University of Helsinki, Helsinki, Finland; k Broad Institute of MIT and Harvard, Cambridge, MA, USA

**Keywords:** Faecal microbiota transplantation, strain engraftment, human gut microbiome, ulcerative colitis, obesity

## Abstract

Disrupted human gut microbiota have been associated with the development of certain disease states, including obesity and ulcerative colitis (UC). Faecal microbiota transplantation (FMT) from healthy donors is a promising avenue to shift the microbiome profile of the recipient towards that of the donor, potentially ameliorating related symptoms. Several recent meta-analyses have investigated the clinical and microbial determinants that influence the retention of transplanted donor microbial strains within the recipient gut microbiome following FMT (i.e. engraftment). However, the specific factors that affect donor strain engraftment in different disease states require further exploration. Here, we perform a strain engraftment analysis on data from two multi-donor FMT clinical trials: the Gut Bugs Trial for obesity and the FOCUS Trial for UC. Using donor strain matching, the donor-recipient pairings of the FOCUS Trial were first predicted in a blinded manner. The subsequent, unblinded, strain engraftment analysis of both datasets highlighted a differential effect of donor-recipient microbiome complementarity on engraftment across the two disease cohorts; greater engraftment efficiency was associated with increased donor-recipient microbial similarity in the FOCUS Trial, and decreased similarity in the Gut Bugs Trial, suggesting that the factors influencing engraftment may differ across disease cohorts.

## Introduction

Faecal microbiota transplantation (FMT) is a therapeutic procedure used to modify disrupted intestinal microbiota through a pre-screened donor stool transplant. At present, FMT is used clinically in the treatment of recurrent *Clostridiodes difficile* infection.^1^ Its use in the treatment of other health conditions that share an element of gut dysbiosis, which may range in nature from inflammatory (e.g. ulcerative colitis (UC)),^2^ to metabolic (e.g. obesity),^3^ neurological (e.g. Parkinson’s disease)^4^ and psychiatric (e.g. bipolar disorder),^5^ is currently being explored through randomised controlled trials^6^^,^^7^ and open label studies. Donor stool samples are screened prior to their selection and application to reduce the potential risk associated with transplanting a consortium of microbes that may contain transient or lowly abundant opportunistic pathogens, or bacterial products that contribute to disease pathogenesis that were non-pathogenic in the donor.^8^ Other aspects of FMT protocols, such as FMT dosage and frequency/timing, are variable across different trials.^7^^,^^9^ For example, FMT therapy may be administered as a single- or multi-donor treatment, in one or multiple doses. The administration route of FMT is also variable, including colonoscopy, nasoenteric tube, capsules or enema, and may be coupled with a bowel cleanse and/or antibiotic pre-treatment.^10^

Two randomised controlled trials, namely the FOCUS Trial^2^^,^^11^ and the Gut Bugs Trial,^3^^,^^12^^,^^13^ explored the efficacy of FMT to address symptoms associated with UC and obesity, respectively. UC is a form of inflammatory bowel disease characterised by inflammation and ulcers in the colon and rectum. The cause of UC is unknown,^14^however, the condition has been associated with perturbations of the gut microbiota.^15^ Evidence of this connection is further strengthened by the convergence of the gut microbiome in patients in remission from UC towards that observed in individuals without the disease.^16^ Consequently, the modulation of the gut microbiome through FMT is an emerging treatment option for UC, with the clinical remission rate at approximately 40%, and endoscopic remission in approximately 17% of patients.^17^ Obesity is a complex metabolic disease characterised by excessive body fat, and associated with an increased risk of comorbidities that include cardiovascular disease, hypertension and type 2 diabetes.^18^ Like UC, obesity is associated with an overall alteration in gut microbiota composition, reflecting a deviation from healthy microbiomes. However, consistent differences in specific taxa have not been identified.^19^ Early studies in mice highlighted the metabolic potential of the gut microbiota, with the transferability of obese and lean phenotypes through the transplantation of associated gut microbiota^20-22^ providing preliminary evidence for the use of FMT as a possible treatment option for people with obesity.

The structural and functional augmentation of the recipient gut microbiome may be facilitated by microbial engraftment, i.e. the long-term retention of novel microbiota that originate from the donor transplant.^13^ Indeed, donor strain engraftment has been positively associated with clinical outcomes in previous FMT studies.^23^^,^^24^ However, this relationship is not absolute, with some studies suggesting that not all donors confer ideal microbiota, and clinical response may depend more on the acquisition of specific beneficial taxa (e.g. butyrate producers) rather than overall engraftment.^25–27^

The increasing availability of clinical FMT trial protocols with different disease targets and their resulting datasets has prompted several recent meta-analyses^10^^,^^23^^,^^28^ to investigate the clinical and microbial determinants of engraftment. Across these studies, the condition of the recipient gut microbial community at the time of FMT treatment was ubiquitously found to influence engraftment.^10^^,^^23^^,^^28^ Specifically, greater gut dysbiosis in FMT recipients (relative to healthy controls) was correlated with increased engraftment.^10^ Moreover, recipient *α*-diversity prior to FMT was inversely correlated with engraftment,^10^which has been proposed as a possible explanation for the observed disparity in engraftment across disease types (increased in infectious compared with non-infectious).^23^ Therefore, the implementation of a pre-treatment bowel cleanse or antibiotic to reduce the endogenous microbial community may subsequently improve the efficacy of donor-mediated microbial modulation across recipients.^10^^,^^23^^,^^28^ Notably, recipient-specific factors that are not modifiable by FMT pre-treatment, such as genetics and immunity, may also influence engraftment.^27^ Other clinical variables that may improve engraftment at the recipient level include the FMT administration route.^23^ Enemas, for example, are limited to the distal end of the gastrointestinal tract, and are therefore relatively ineffective at FMT distribution.^29^ A mixture of FMT routes^23^ and multiple FMT applications^10^ appear to be positively associated with engraftment, presumably due to their facilitation of greater microbial colonisation.

The engraftment potential of specific donor microbial strains is dependent on their phenotype and environmental niche,^10^^,^^23^as well as their complementarity with available niches within the recipient microbial community.^28^ Species with greater differential abundance (higher in donor, lower or lacking in recipient) will be more likely to engraft,^10^ as this implies niche availability. Furthermore,species strain richness has recently been shown to influence engraftment dynamics following FMT.^30^ Optimising the engraftment of species that have been associated with improved treatment outcomes can therefore be considered a recipient-specific endeavour, with tailored combinations of donor(s) and recipients ultimately required to realise the potential of FMT as personalised medicine. Nonetheless, it has been noted that engraftment may have a large element of stochasticity and therefore be limited in its prediction, even under optimum conditions.^28^

Typically, published clinical FMT trial datasets will provide metagenomic sequencing data for donors and recipients (pre- and post-treatment), in addition to details of the donor-recipient pairings (for multi-donor FMT trials). This information is vital for tracking how the recipient gut microbial composition has been modulated by the donor transplant. In some cases, however, FMT donor-recipient pairings may be withheld to protect participant privacy or for other reasons associated with data sensitivity. In these cases, it may still be important to be able to assess donor contributions in the absence of reported donor-recipient pairings, in order to understand the interactions between donor and recipient microbiota and the correlation with treatment success. This information could then be used to screen and assign FMT donors in a bespoke manner in future FMT trials for a given health condition. Here, we explore an approach utilising donor strain matching (strain engraftment analysis) to predict donor-recipient pairings, using data from a multi-donor FMT trial (FOCUS Trial) for patients with UC.^2^^,^^11^ We validate the analysis using a second multi-donor FMT trial (Gut Bugs Trial) for people with obesity.^3^^,^^12^^,^^13^ We also investigate how the interaction of donor and recipient microbiomes explains differences in the recall of different donors based on strain engraftment.

## Methods

### Data acquisition

Metagenomic sequencing files were obtained from a published double-blind, randomised, placebo-controlled clinical FMT trial (FOCUS Trial).^2^^,^^11^ The trial protocol comprised a single colonoscopic FMT infusion, followed by 40 enema treatments over a period of 8 weeks. The FMT donor stool was pre-screened for enteric pathogens and parasites. Bowel preparation was performed prior to the initial colonoscopy. Participants were not administered antibiotics as part of the pre-treatment. The placebo recipients received a placebo masked with colourant and odorant. Each FMT recipient received multi-donor FMT from a fixed group of 4−7 donors (total *n* = 14), which varied across the recipient cohort. Stool samples were obtained from the participants (FMT and placebo) at the baseline screening and every four weeks during the blinded treatment phase. Placebo participants were subsequently offered open-label FMT, with further samples taken eight weeks following the open-label treatment, where applicable. Samples were also obtained from the individual donors and the pooled donor batches administered to the recipients. Metagenomic paired-end sequencing data was publicly available for 284 samples. Of these, all FMT and placebo recipient samples from baseline and week 8, individual donor and donor batch samples were selected for this analysis (total *n* = 157) (Supplementary File 1). Metagenomic sequencing data for placebo recipients at week 4 were not available. Therefore, the sample subset was selected to capture all donor samples, as well as recipients at the available pre- and immediate post-FMT timepoints. A total of 32 FMT recipients and 20 placebo recipients had samples available at baseline and 8 weeks post-intervention.

The analysis of the FOCUS Trial samples was initially performed blind, i.e. without knowledge of FMT donor-recipient pairings. Following the prediction of the FMT donor-recipient pairings, the true pairings were acquired from the FOCUS Trial study authors, and these were used in subsequent analyses. The point at which the donor-recipient pairings were acquired is indicated in the methods, all methods prior to this point were conducted blind.

To validate the analysis, metagenomic sequencing files were obtained from a second double-blind, randomised, placebo-controlled clinical FMT trial (Gut Bugs Trial).^3^^,^^12^^,^^13^ Metagenomic sequencing paired-end data was available for 381 samples, with an average of 45.7 ± 6.1 million microbial reads per sample (mean ± SD). The intervention was administered to recipients over two consecutive days as 28 double encapsulated acid-resistant capsules, with each FMT recipient receiving a multi-donor FMT from four sex-matched donors (total *n* = 9). The FMT donor stool was pre-screened for faecal pathogens and parasites. Placebo recipients received capsules containing saline solution instead of the active treatment. Similar to the FOCUS Trial, the Gut Bugs Trial participants underwent bowel cleansing prior to treatment, with no antibiotic pre-treatment. Stool samples were obtained from the participants (FMT and placebo) at the baseline screening, and 6- 12- and 26 weeks following the intervention. Multiple samples were also obtained from the FMT donors throughout the stool donation period. From the 381 samples available, 228 samples were selected to capture all FMT and placebo recipient samples from baseline and week 6, and all donor samples, to match the FOCUS Trial data subset as closely as possible (Supplementary File 2). A total of 39 FMT recipients and 44 placebo recipients had samples available at baseline and 6 weeks post-intervention. As the true Gut Bugs Trial FMT donor-recipient pairings were already published at the time of this study, a blinded analysis of donor strain engraftment was not able to be performed on this trial. All data analysis was performed in R (version 4.4.0),^31^using tidyverse packages (version 2.0.0).^32^ Figures were produced using ggplot2 (version 3.5.1),^33^ unless otherwise specified.

### Bioinformatic pre-processing

For each trial, sequencing files were processed using the bioBakery platform, run on the cloud-native platform Terra.^34^^,^^35^ Pre-processing of the FOCUS Trial sequencing files to remove low-quality and human reads was performed using KneadData (version 0.10.0) and the human genome reference hg37.^36^ The Gut Bugs Trial sequencing files had been quality-controlled and processed with KneadData prior to their acquisition.^13^

### Taxonomic profiling

Taxonomic profiling of post-processed reads was performed using MetaPhlAn3 (version 3.1) while accounting for the abundance of sequences of unknown origin.^37^

### Blinded taxonomic profiling (FOCUS Trial)

Bacterial species *α*-diversity (Shannon’s diversity index) was calculated for each individual donor sample of the FOCUS Trial (R vegan package version 2.6.6.1). Donor *α*-diversity was compared using an ANOVA test (R stats package version 4.4.0). Post-hoc analysis was performed using the Tukey Honest Significant Differences test (R stats package version 4.4.0) to identify donors with significantly different *α*-diversity. To visualise differences between the microbiome profiles (*β*-diversity) of recipients (FMT and placebo) and donors (batch and individual) at baseline and week 8, a principal coordinate analysis was performed on all samples using the Bray-Curtis dissimilarity index. Recipient samples were subset to visualise differences at baseline and week 8, relative to the donor samples. To assess these differences statistically, a permutational analysis of variance (PERMANOVA) was performed with 999 permutations on species relative abundance data, comparing all recipients at baseline with individual donor samples. PERMANOVA was performed using mean species relative abundance values for individual donors, where more than one sample was available. Donor batch values were not included in the PERMANOVA. Additional PERMANOVAs (999 permutations) were performed to assess the effect of recipient treatment group on *β*-diversity at baseline and week 8 (adonis2, R vegan package, version 2.6.6.1). The *Prevotella*/*Bacteroides* ratio of each individual donor sample was calculated from the MetaPhlAn3 relative abundance data for the respective genera. Donor *Prevotella*/*Bacteroides* ratios were compared using the Kruskal-Wallis rank sum test (R stats package version 4.4.0). Post-hoc analysis was performed using the Dunn test (dunn.test package version 1.3.6) to identify donors with significantly different *Prevotella*/*Bacteroides* ratios.

### Strain profiling

For each sample in each trial, the dominant strain of each species with sufficient relative abundance was profiled using StrainPhlAn3 (MetaPhlAn3, version 3.1).^38^ This approach was previously published by Wilson et al.^13^ Strain profiling was performed on Terra, using the StrainPhlAn3 default settings. For each species, strain phylogeny was estimated from pairwise comparisons across all samples using the R package phangorn (version 2.11.1),^39^ which calculates DNA similarity distances using the Jukes and Cantor (JC69) model. DNA similarity distances were subsequently normalised by the median distance of all pairwise comparisons for each species, as different species may exhibit different levels of strain diversity.

### Blinded donor strain engraftment analysis (FOCUS Trial)

**Figure 1.**
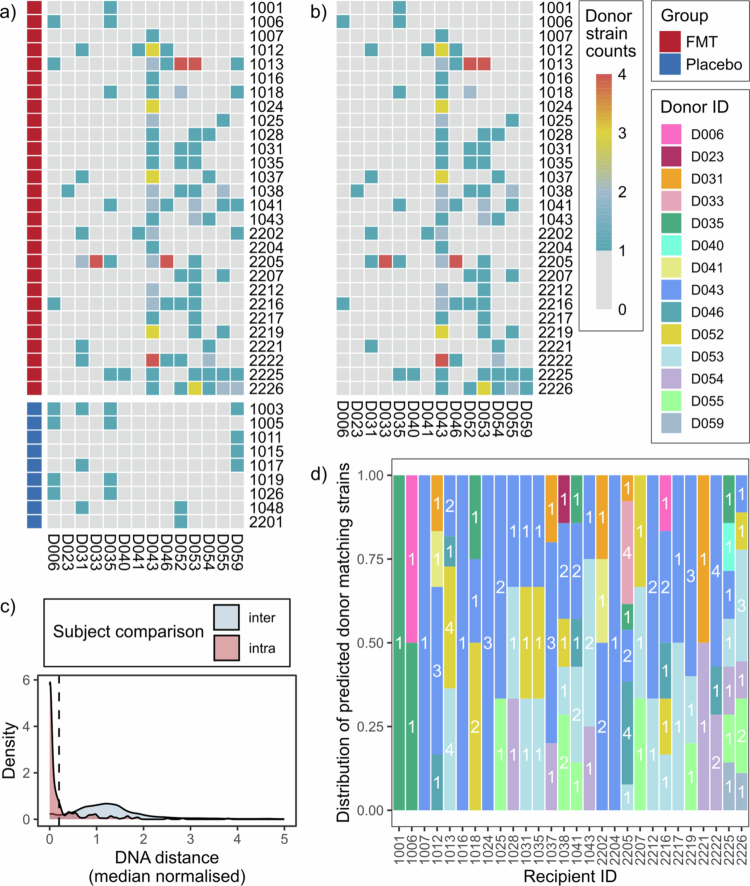
Predicted donor profile for each FOCUS Trial FMT recipient with donor-matching strains. (a): Novel donor-matching strains (i.e. not present at baseline) identified from a stringent normalised DNA distance threshold of 0.2 are shown for FMT recipients (above; dark red identified rows) and placebo recipients (below; dark blue identified rows) at 8 weeks post-intervention. (b): Ambiguous donor strains identified in placebo recipients were removed from the FMT data, and the number of donor-matching strains at week 8 was re-plotted. The heatmap scale represents the number of predicted engraftment events from each donor (columns) to each recipient (rows) involving distinct species strains. Only recipients with donor-matching strains are shown (FMT *n* = 28/32, placebo *n* = 9/20). (c): The density of DNA distance (median normalised) values for strains from inter- (grey) and intra-subject (pink) pairwise comparisons is plotted. Donor batch samples were excluded from pairwise comparisons due to their ambiguity as intra- or inter-subject comparisons. Total pairwise comparisons considered = 40,052. Pairwise comparisons ≥5 were not plotted due to their low density. Strains with ≤0.2 normalised DNA distance (dashed line) are considered a strain match. (d): Novel donor-matching strains unique to the FMT recipient cohort were identified in 28/32 FMT recipients at week 8. The distribution of donors with matching strains was plotted to assign a tentative donor profile for each of these FMT recipients (range: 1−7 donors per recipient). Bars are coloured by FMT donors. Count data and bar height correspond to the number of distinct species strains matching each donor in each FMT recipient sample at week 8. FMT, faecal microbiota transplantation.

A consensus threshold for microbial strain identity is yet to be determined.^23^ Therefore, to establish a threshold for strain similarity in the blinded analysis of the FOCUS Trial data, the distributions of normalised DNA distance for strains within each participant (i.e. samples from the same donor, or recipient samples at different timepoints) were compared with those from between participants (e.g. donor and recipient) using a density plot. The visualisation of strain DNA distance within and between participants produced two distinct peaks (Figure 1c). To predict donor recipient strains of donor origin, a normalised DNA distance threshold of ≤0.2 was selected, which captured most genetically-similar (intra-participant) strains whilst excluding most genetically-distinct (inter-participant) strains, as was previously demonstrated in strain matching analyses for the Gut Bugs Trial.^13^^,^^40^ Therefore, any strain with a normalised DNA distance less than 0.2 in a pairwise sample comparison was considered a strain match. Donor-matching strains within recipient week 8 samples were excluded if they were present in a recipient at baseline, as these were considered to be of recipient origin. Similarly, donor strain matches that were present in both FMT and placebo recipients were subsequently removed as background due to their ambiguous nature. Distinct donor species strain matches were plotted with the R package pheatmap (version 1.0.12). The number of donor-matching strains in each recipient was used to predict the group of donors for each FMT recipient. The number of distinct species strains matching each donor was quantified for each FMT recipient and plotted to visualise the predicted donor pools.

To understand whether the predicted widespread engraftment of strains from donor D043^2^^,^^11^ across FMT recipients was driven by a single strain species with superior engraftment, the strain match data was filtered for D043-specific events. The number of different engrafted species matching either of D043’s samples were quantified.

### Unblinded donor strain engraftment analysis

The true FMT donor-recipient pairings were obtained from the FOCUS Trial study authors^2^^,^^11^ on 15/05/2024 (Supplementary Figure 1), and were used in the analysis from this point onwards. The following analyses were also performed on data from the Gut Bugs Trial.^3^^,^^12^^,^^13^

### Selection of optimal DNA distance threshold

For each trial, the FMT-recipient pairings predicted using the initial normalised DNA distance threshold of 0.2 were compared to the true pairings, to obtain an F1 score. This step was repeated for all normalised DNA distance thresholds between 0.001 and 3.000, which had the highest density of pairwise comparisons in the FOCUS Trial (Figure 1c) and Gut Bugs Trial (Supplementary Figure 2), to obtain the normalised DNA distance thresholds that predicted the true pairings with the highest precision and recall. FMT donor-recipient pairings were subsequently predicted through strain engraftment analysis, using the optimal normalised DNA distance threshold. For each FMT recipient, the number of distinct species strains matching each donor were quantified and plotted to visualise the predicted donors for each recipient.

### Comparison of FMT donor recall and engraftment efficiency

FMT donor recall (i.e. true positive rate) was calculated as the proportion of true positive recipient pairings correctly predicted for each donor. FMT donor engraftment efficiency was calculated as the proportion of distinct donor strains (pooled across each donor’s samples) detected in each of their true recipients. Donor engraftment efficiency was compared using the Kruskal-Wallis rank sum test (R stats package version 4.4.0). Post-hoc analysis was performed using the Dunn test (R dunn.test package version 1.3.6) to identify donors with significantly different engraftment efficiency. The correlation of recall and engraftment efficiency across all donors was assessed using a linear model and the strength of fit was assessed using Pearson’s correlation.

To further understand strain engraftment dynamics, the number of distinct engrafted strain species that were present in their respective FMT recipient baseline samples was quantified. Engrafted species present within the recipient microbiome at baseline were considered to have replaced the existing conspecific recipient strain. Engrafted species absent from the recipient microbiome at baseline were considered novel to the recipient. The proportion of novel engrafting strains was compared between the FOCUS Trial and Gut Bugs Trial cohorts using a proportion test (R stats package version 4.4.0).

### Characterisation of factors underlying donor engraftment efficiency

To understand whether the intersection of donor and recipient *α*-diversity influenced engraftment efficiency, Shannon’s diversity index was calculated for each FMT recipient baseline sample and the mean Shannon’s diversity index was calculated across each FMT donor’s samples (R vegan package version 2.6.6.1). The respective *α*-diversity values for each donor-recipient pairing were plotted as a scatterplot, with the point size corresponding to the engraftment efficiency of that pairing. Mean donor and FMT recipient baseline *α*-diversity values were compared using the Wilcoxon rank sum test (R stats package version 4.4.0). The correlation of donor engraftment efficiency and donor *α*-diversity was assessed using a linear model and the strength of fit was assessed using Pearson’s correlation.

To visualise whether donor and recipient microbiome dissimilarity (*β*-diversity) was associated with engraftment efficiency, the species *β*-diversity of all individual donor and FMT recipient baseline samples was calculated using the Bray-Curtis dissimilarity index and Jaccard dissimilarity index, with the vegan ‘vegdist()’ function (version 2.6.6.1). When calculating the Jaccard dissimilarity index, the option ‘binary = TRUE’ was used. The correlation of engraftment efficiency with all donor-recipient (baseline) pairing *β*-diversity values was assessed using a linear model for each *β*-diversity measure, and the strength of fit was assessed using Pearson’s correlation. For donors with multiple samples, the mean microbiome dissimilarity to each of their recipients was calculated for each *β*-diversity measure.

To understand any association between donor and recipient microbiome functional dissimilarity (*β*-diversity) and engraftment efficiency, functional annotations were first obtained for genes on high-quality metagenome-assembled genomes (MAGs) (i.e. >90% completeness and <5% contamination). MAG construction included the assembly of sequencing reads into contigs of a minimum length of 500 base pairs, using MEGAHIT (version 1.1.4).^41^ Contig sequences were then binned to produce MAGs using MetaBAT 2 (version 2.15−3).^42^ Eight samples of the FOCUS Trial with the lowest QC read counts were unable to be binned (Supplementary File 3), and were therefore excluded from further steps utilising MAG sequences. MAG completeness and contamination were assessed with CheckM (version 1.1.2).^43^ Gene prediction from contig sequences was performed using Prodigal (version 2.6.3).^44^ Genes were clustered with a >95% identity threshold using cd-hit-est (version 4.7) to produce a non-redundant gene catalogue.^45^ Functional annotations were generated for clustered genes on high-quality MAGs using eggNOG (version 2.0.1).^46^

Across FMT donor and baseline recipient samples, there were 386,865 high-quality genes with MAG membership in the FOCUS Trial and 2,882,813 in the Gut Bugs Trial. The eggNOG functional annotations obtained for the high-quality genes included GO,^47^^,^^48^KEGG^49-51^ and COG.^52^^,^^53^ Of these tools, COG provided the most annotations with 86.1% (333,194/386,865) and 86.3% (2,488,193/2,882,813) of these subsets, respectively, gaining a COG functional category annotation. Thus, to achieve the most comprehensive understanding of the influence of donor-recipient functional dissimilarity on engraftment efficiency, the COG functional profiles of donors and their recipients at baseline were compared. The relative abundance of each COG functional category per sample was calculated as a proportion of the number of high-quality genes with MAG membership in that sample. Genes without COG functional category annotations were assigned NA. Genes with multiple COG functional category annotations were counted separately. The relative abundance data was subsequently renormalised, with the sum of the relative abundances in each sample equalling one. The functional profile *β*-diversity of all individual donor and FMT recipient baseline samples was calculated using the Bray-Curtis dissimilarity index. The correlation of engraftment efficiency with all donor-recipient (baseline) pairing functional *β*-diversity values was assessed using a linear model, and the strength of fit was assessed using Pearson’s correlation. For donors with multiple samples, the mean microbiome functional dissimilarity to each of their recipients was calculated. Eleven samples from the FOCUS Trial had no high-quality genes and were not able to be plotted (Supplementary File 4). Jaccard dissimilarity index comparisons for functional profiles were excluded due to most samples having identical profiles.

### Identifying factors most predictive of engraftment efficiency

To test which factors were the most predictive of engraftment efficiency, linear mixed models were fitted against the engraftment efficiency data using the R package lme4 (version 1.1.35.3), with the package lmerTest (version 3.1.3) used to obtain *p* values. For each donor-recipient pairing, the donor engraftment efficiency was calculated and data was collated for the following metrics: mean donor sample *α*-diversity (Shannon index), recipient baseline sample *α*-diversity (Shannon index), mean donor-recipient (baseline) species *β*-diversity (Bray-Curtis dissimilarity index and Jaccard dissimilarity index), mean donor sample *Prevotella*/*Bacteroides* ratio, recipient baseline sample *Prevotella*/*Bacteroides* ratio, mean donor-recipient (baseline) COG functional category *β*-diversity (Bray-Curtis dissimilarity index), and total number of donors in the FMT batch (FOCUS Trial only). The above metrics were considered fixed effects. Within donor variations and within recipient variations were considered random effects. After comparing the fit of the full (interactions between donor and recipient *α*-diversity, and between donor and recipient *Prevotella*/*Bacteroides* ratio) and reduced (no interactions between the fixed effects) models using a likelihood ratio test in the base R stats package (version 4.4.0) (FOCUS Trial *p* = 0.021; Gut Bugs Trial *p* = 0.097), all interactions were included in the FOCUS Trial model, but were excluded from the Gut Bugs Trial model. The fixed effect estimate sizes were extracted using the lme4 function ‘fixef()’ and plotted. The fixed effect sizes from both the reduced and full models were plotted for each trial to enable direct comparisons across the datasets. However, the results were only reported for the best fitting model for each trial.

To further identify whether the difference in donor-recipient abundance of individual COG functional categories was predictive of donor-recipient engraftment efficiency in cases where the above linear mixed model had identified a significant effect of mean donor-recipient (baseline) COG functional category Bray-Curtis *β*-diversity on engraftment efficiency (FOCUS Trial only), an additional linear mixed model was fitted against the data. The mean difference in normalised relative abundance of each COG functional category in the donor samples relative to their recipient’s baseline sample was calculated and these were used as the fixed effects. Donor and recipient IDs were used as random effects to correct for other donor-recipient effects unaccounted for by the metadata.

It is possible that the observed significant effect of functional and species *β*-diversity profiles on engraftment efficiency may have been an artefact of sequencing depth. To assess this, principal coordinate analyses were performed on the renormalised COG functional relative abundance data and the normalised species relative abundance data, respectively, for all individual donor and FMT recipient samples, to capture the sample set used to assess the impact of *β*-diversity on FMT donor-recipient engraftment efficiency. The *β*-diversity profiles were calculated using the Bray-Curtis dissimilarity index, and the Jaccard dissimilarity index using the option ‘binary = TRUE’ which first converted the relative abundance data to presence/absence values. The KneadData post-QC total read counts for each sample were quantified as the sum of the paired and orphan reads. PERMANOVAs were performed with 999 permutations, comparing the total read count of each sample with the functional or species relative abundance data (Bray-Curtis), or presence/absence values (Jaccard) (R vegan package, version 2.6.6.1). Differences in *β*-diversity profiles due to read count were visualised using a principal coordinate analysis.

## Results

### Blinded prediction of FMT donor-recipient pairings

The FOCUS Trial utilised a multi-donor FMT treatment, with stool from 4−7 different donors combined to form the treatment for each recipient with UC.^2^^,^^11^ Metagenomic sequencing data from stool samples consisted of, on average, 4.4 million ± 2.0 million post-QC reads per sample (mean ± SD).

To predict FMT donor-recipient pairings from metagenomic sequencing data, we used an approach predicated on the assumption that if a bacterial strain novel to the post-FMT recipient microbiome was genetically matching a strain in a donor microbiome, the source of the strain is the donor, and therefore that donor was used as part of the multi-donor batch treatment for that recipient. Strain profiling produced a total of 1,181 individual strain profiles, belonging to 36 different species. An average of 8 strains (range: 1−17) were identified per sample. To determine a threshold of strain similarity, the genetic similarity of strains within and between participant samples was compared (Figure 1c). In an effort to favour false negative predictions over false positives, a median normalised DNA distance threshold of 0.2 was selected, as described previously.^13^^,^^40^

Using this strain matching threshold, donor-matching strains were detected in 87.5% (28/32) of FMT recipients and 45% (9/20) of placebo recipients (Figure 1a), suggesting not all of these events could be related to FMT engraftment. To reduce the ambiguous donor matches, specific donor strains with matches in both FMT and placebo recipients were subsequently removed (Figure 1b), with the number of FMT recipients with donor strain matches remaining unchanged. From this data, donors were tentatively assigned for each FMT recipient (range: 1−7 donors per recipient), where donor-matching strains were detected (Figure 1d).

### Donor D043 was the predominant origin of predicted engrafted strains across recipients

**Figure 2.**
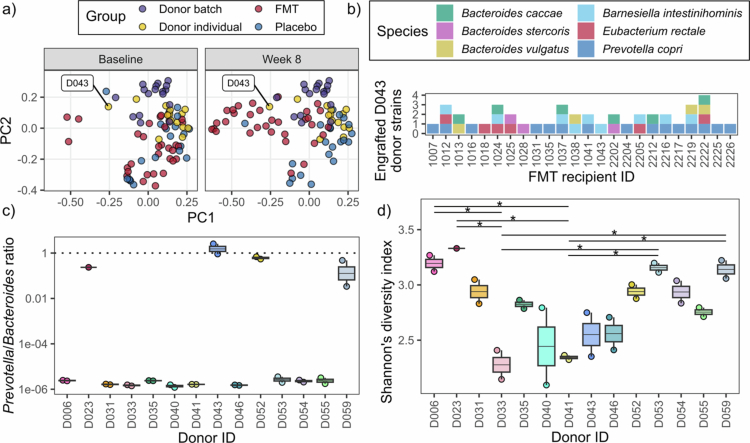
FMT recipient microbiome profiles shifted towards D043 following treatment. (a): Classical (metric) multidimensional scaling of species relative abundance in FMT and placebo recipient samples at baseline and 8 weeks post-intervention using the Bray-Curtis dissimilarity index, compared to individual donor and donor batch samples. Each point represents a sample. Where a single donor (individual or batch) had multiple samples, the mean PC1 and PC2 coordinates were plotted. (b): D043 species strain matches in FMT recipients at week 8. (c): *Prevotella*/*Bacteroides* ratio and (d) Shannon’s diversity index values for individual donor samples. Boxes represent the interquartile range (IQR) split by the median, with whiskers extending up to 1.5x the IQR. FMT, faecal microbiota transplantation; PC1, principal coordinate 1; PC2, principal coordinate 2; *p < 0.05.

Following the prediction of donor profiles for each FMT recipient, classical multidimensional scaling of species relative abundances was performed to visualise the variance of bacterial species following FMT treatment. At baseline, recipient microbiomes were compositionally distinct from individual donor profiles (PERMANOVA, *p* < 0.001, *R*² = 0.040), but were not different between treatment groups (PERMANOVA, *p* = 0.847, *R*² = 0.014). At 8 weeks post-intervention, FMT microbiomes were compositionally distinct from placebo microbiomes (PERMANOVA, *p* < 0.001, *R*² = 0.120) and appeared to have shifted towards one donor profile: D043 (Figure 2a). D043 strain engraftment was linked to 6 different species strains, including *Prevotella copri* (*Segatella copri*) that were detected in 47% (15/32) of FMT recipients at week 8 (Figure 2b). Across all donors, D043 was the only donor with an average *Prevotella*/*Bacteroides* ratio greater than 1 (i.e. *Prevotella* dominance) (Figure 2c), an attribute that has been previously linked with the super-donor effect (i.e. a donor with superior engraftment relative to other donors).^13^ Donor *Prevotella*/*Bacteroides* ratios were significantly different (Kruskal-Wallis rank sum test, *p* = 0.029). However, post-hoc testing did not identify any pairwise significant differences after adjusting *p* values for multiple testing. Conversely, the *α*-diversity of D043 samples was relatively low when compared with other donors, an attribute that was positively correlated with bacterial engraftment in another study.^10^ Across all donors, *α*-diversity was significantly different (ANOVA, *p* = 0.002), with post-hoc testing identifying pairwise significant differences (Tukey test) (Figure 2d). It is therefore unclear whether the predominance of D043 strain matches across the FMT recipient cohort is a consequence of their increased use in the FOCUS Trial batch treatments, or indicates superior engraftment outcomes compared to other donors.

### Evaluation of blinded FMT donor-recipient pairing predictions

**Figure 3.**
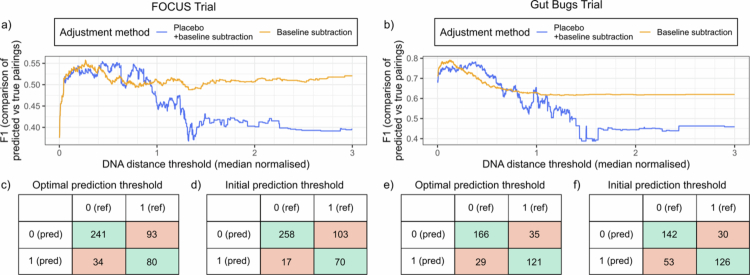
Optimised strain matching thresholds have greater F1 scores than initial thresholds. The F1 score was calculated for DNA distance thresholds between 0.001−3, at intervals of 0.001 for (a) the FOCUS Trial and (b) the Gut Bugs Trial, by comparing the respective predicted FMT donor-recipient pairings with the true (reference) pairings. By default, donor strain matches in FMT recipients at the first post-intervention timepoint (FOCUS Trial: week 8; Gut Bugs Trial: week 6) were only considered to be novel if the strain was absent from the recipient at baseline (baseline subtraction). An additional step to remove donor strains with matches in the placebo cohort was also performed (placebo + baseline subtraction). In (a), thresholds ≥0.002 are plotted, to remove the initial long tail. The confusion matrices for the FOCUS Trial (c) optimal (0.267) and (d) initial (0.2), and the Gut Bugs Trial (e) optimal (0.123) and (f) initial (0.2) strain matching thresholds used for donor strain engraftment analysis are also shown. FMT, faecal microbiota transplantation; ref, reference; pred, predicted.

The true FMT donor-recipient pairings used in the FOCUS Trial were obtained on 15/05/2024 (Supplementary Figure 1, Supplementary Figure 3), and were used for validating the predicted FMT donor-recipient pairings. To evaluate the efficacy of using matching donor strains to predict FMT donor-recipient pairings in a blinded manner, the F1, precision and recall scores were quantified for the predicted FOCUS Trial pairings (Figure 1d) that were obtained using a strain matching threshold of 0.2 and placebo adjustment (i.e. identifying only donor-matching strains unique to the FMT recipient cohort). The F1 score was 0.538 (Figure 3a), and the model achieved a precision of 0.805 and a recall of 0.405.

### Gut microbiota profiles differed between the FOCUS Trial and Gut Bugs Trial cohorts

To validate the unblinded analysis of the FOCUS Trial dataset, a similar analysis was repeated with an additional FMT dataset from the Gut Bugs Trial for obesity.^3^^,^^12^^,^^13^ The Gut Bugs Trial was a double-blind randomised placebo-controlled FMT trial, with metagenomic sequencing data available for 9 donors, and 39 FMT recipients and 44 placebo recipients sampled at both baseline and 6 weeks post-intervention. Each FMT recipient received a stool transplant from four sex-matched donors. Additional sample data was available for FMT and placebo recipients at 12- and 26 weeks post-FMT, however, these were not required for this analysis. Across the whole dataset, there was an average of 45.7 million microbial reads per sample^13^; approximately 10 times greater than the sequencing depth used in the FOCUS Trial.^11^ For this dataset, it was not possible to perform the analysis in a blinded manner, due to prior knowledge of the true FMT donor-recipient pairings (Supplementary Figure 3b).

While a number of different health conditions with an element of gut microbial dysbiosis are currently being explored as potential treatment targets for FMT, gut microbiota have previously been shown to display different signatures depending on the disease cohort.^54^ To visualise differences in the gut microbiota of people with UC and obesity, a principal coordinate analysis was performed on the species *β*-diversity in all recipient (FMT and placebo) baseline samples. There was a significant difference in *β*-diversity between the FOCUS Trial and the Gut Bugs Trial samples (PERMANOVA, *p* < 0.001, *R*² = 0.063) (Supplementary Figure 4), consistent with differences in microbial profiles or technical differences between the two cohorts.

### Selection of optimal thresholds for strain engraftment analysis

To understand the variability in donor engraftment across FMT recipients, an optimal strain matching threshold was first selected. The original strain-based analysis of the Gut Bugs Trial dataset utilised an equal strain matching threshold of 0.2.^13^ Most normalised DNA distance values obtained from the pairwise strain matching for both trials were between 0−3 (Figure 1, Supplementary Figure 2). Therefore, the distribution of predicted donors for each recipient produced by strain matching thresholds within this range were tested against the true distribution of predicted donors, to determine the strain matching threshold with the greatest precision and recall (Supplementary Figure 5). For the FOCUS Trial, the optimal strain matching threshold was 0.267, which had an F1 score of 0.557 (Figure 3a), and predicted ten more true positive donor-recipient pairings compared with the previously used threshold of 0.2 (Figure 3c,d). The optimal strain matching threshold identified for the Gut Bugs Trial was 0.123, which had an F1 score of 0.791 (Figure 3b). While five fewer true positive pairings were predicted using this threshold compared with the threshold used in the original analysis (Figure 3e,f), there were also 24 more true negatives identified. The strain engraftment results produced with the optimal strain matching thresholds were subsequently used for the remainder of the analyses presented in this study.

Using the optimal strain matching normalised DNA distance threshold of 0.267 with no removal of donor strains also matching the placebo cohort, FMT donor-recipient pairings were predicted for the FOCUS Trial (Supplementary Figure 6a), with donor-matching strains detected in 90.6% (29/32) of FMT recipients. The prediction of donor-recipient pairings was also performed for the Gut Bugs Trial using the optimised threshold of 0.123, with no removal of donor strains also matching the placebo cohort (Supplementary Figure 6b). Donor-matching strains were detected in 100% (39/39) of FMT recipients. The true pairings used in each trial are available for reference (Supplementary Figure 3).

### Recall and engraftment efficiency were variable across donors

On average, FMT donors were correctly predicted for a recipient in 41.7% of pairings in the FOCUS Trial (donor recall range: 0−83%) (Supplementary Figure 7a) and 79.7% of pairings in the Gut Bugs Trial (donor recall range: 29−100%) (Supplementary Figure 7b). Given the varied use of FMT donors in the FOCUS Trial (range: 1−29 recipients) (Supplementary Figure 3a), we tested whether the ability to correctly predict FMT donors was influenced by their usage frequency. There was no significant correlation between donor recall and the number of FMT recipients of each FOCUS Trial donor (Pearson’s correlation, *p* = 0.370, *R*² = 0.067) (Supplementary Figure 7c).

**Figure 4.**
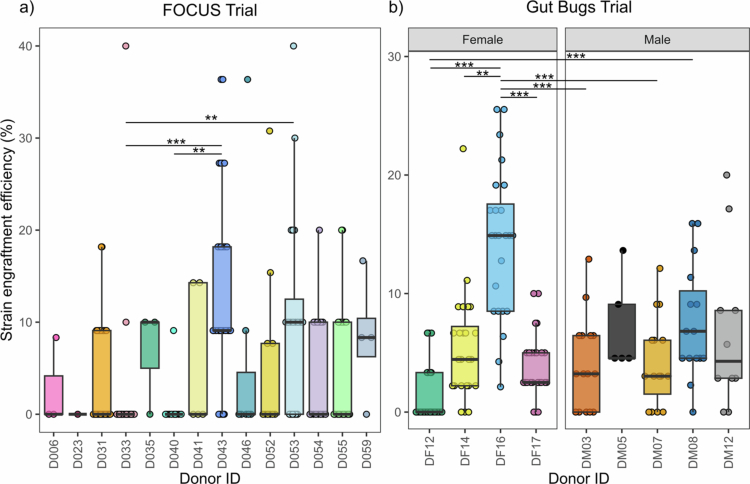
Engraftment efficiency varied across donors within each FMT trial. Mean strain engraftment efficiency was calculated as the mean percentage of distinct donor strains engrafted in their true recipients, for each FMT donor in (a) the FOCUS Trial and (b) the Gut Bugs Trial. Only significant pairwise comparisons where *p* < 0.01 following post-hoc testing (Dunn test) are shown. FMT, faecal microbiota transplantation; **p < 0.01; ***p < 0.001.

The engraftment efficiency of donors, i.e. the number of distinct donor strains found to engraft in their recipients, was also variable in both the FOCUS Trial (Kruskal-Wallis rank sum test, *p* < 0.001) (Figure 4a) and Gut Bugs Trial (Kruskal-Wallis rank sum test, *p* < 0.001) (Figure 4b). Despite the improved detection of distinct donor-matching species strains in the Gut Bugs Trial (*n* = 57; FOCUS Trial *n* = 19), the ranges of mean donor engraftment efficiency were similar (FOCUS Trial: 0−14.1%; Gut Bugs Trial 1.4−14.4%). Post-hoc pairwise comparisons of donor engraftment efficiency in the FOCUS Trial identified D043 and D053 as donors with high engraftment efficiency, while D033 and D040 had low engraftment efficiency. In the Gut Bugs Trial, DF16 had significantly higher engraftment efficiency than five other FMT donors, while DF12 had significantly lower engraftment efficiency than three other FMT donors. Across the FMT recipient cohorts, most engrafted donor strains were novel species to the recipient gut at baseline (FOCUS Trial: 89/124; Gut Bugs Trial: 235/381). The proportion of novel engrafting strains did not significantly differ between trial cohorts (proportion test, *p* = 0.054).

We hypothesised that the ability to recall each donor using strain matching would be influenced by donor engraftment efficiency. To test this, we performed a correlation analysis of these two metrics, for each trial. Across the donor cohort of each trial, donor recall was correlated with engraftment efficiency. This correlation was stronger in the FOCUS Trial (Pearson’s correlation, *p* < 0.001, *R*² = 0.817) (Supplementary Figure 8a), compared with the Gut Bugs Trial (Pearson’s correlation *p* = 0.028, *R*² = 0.524) (Supplementary Figure 8b). This finding suggests that the accurate prediction of donor-recipient pairings using strain matching is influenced by the proportion of donor strains that are engrafted in each recipient.

### Determinants of donor engraftment efficiency

To understand why FMT donors in each trial exhibited significantly variable engraftment efficiency, a number of possible factors were considered. The diversity within a microbial community (e.g. the human gut microbiome) is also known as its *α*-diversity. One commonly used metric for measuring *α*-diversity and comparing values between communities (samples) is Shannon’s diversity index,^55^ which considers both species richness (i.e. the number of different species in a sample) and species evenness (i.e. how consistently different species are represented in a sample). In their meta-analysis, Podlesny and colleagues observed that donor *α*-diversity had a positive effect on engraftment, while recipient pre-treatment *α*-diversity had a negative effect.^10^ Therefore, we expected that engraftment efficiency would be the highest for FMT donor-recipient pairs with the greatest *α*-diversity disparity, prior to treatment. In this analysis, a mean donor *α*-diversity value of 2.5 was an apparent threshold for effective engraftment, with 86.5% (32/37) of donor-recipient pairings in the FOCUS Trial with donor *α*-diversity values lower than 2.5 having 0% engraftment efficiency, compared with only 44.9% (61/136) of pairings with donor *α*-diversity values greater than 2.5 (Supplementary Figure 9a). The mean *α*-diversity of all donors in the Gut Bugs Trial exceeded 2.5 (Supplementary Figure 9b). Donor *α*-diversity was greater in the FOCUS Trial donors than in the respective FMT recipients at baseline (Wilcoxon rank sum test, *p* = 0.004) (Supplementary Figure 10a), which is consistent with the reduced microbial diversity observed in patients with UC.^16^ Nonetheless, across all FMT donors in the FOCUS Trial, mean donor *α*-diversity was not significantly correlated with engraftment efficiency (Pearson’s correlation, *p* = 0.955, *R*² = 2.83 × 10^−04^) (Supplementary Figure 11a). Reduced microbial diversity has previously been associated with obesity in some study cohorts.^19^ However, in this study, the Gut Bugs Trial FMT donor and recipient baseline *α*-diversity values were not significantly different (Wilcoxon rank sum test, *p* = 0.567) (Supplementary Figure 10b). There was also no significant correlation between mean donor strain engraftment efficiency and mean donor *α*-diversity in the Gut Bugs Trial (Pearson’s correlation, *p* = 0.099, *R*² = 0.341) (Supplementary Figure 11b), however, this may be a consequence of relatively small sample sizes.

**Figure 5.**
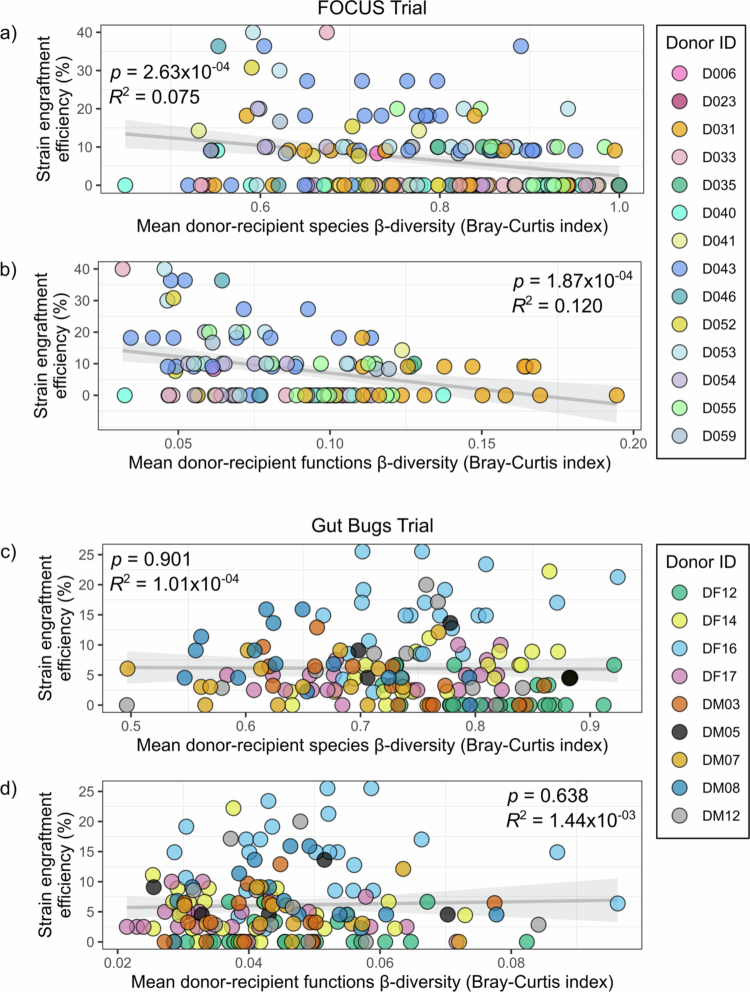
The correlation between donor-recipient species- or functional *β*-diversity (Bray-Curtis dissimilarity index) and engraftment efficiency differed between the trials. The correlation between strain engraftment efficiency and mean donor-recipient (baseline) species Bray-Curtis *β*-diversity was plotted using a linear model for (a) the FOCUS Trial and (c) the Gut Bugs Trial. Species profiles were obtained from MetaPhlAn3. Points are coloured by FMT donors. The grey shaded region represents the 95% confidence interval. FOCUS Trial degrees of freedom = 171; Gut Bugs Trial degrees of freedom = 154. The correlation between strain engraftment efficiency and mean donor-recipient (baseline) functional Bray-Curtis *β*-diversity was plotted using a linear model for (b) the FOCUS Trial and (d) the Gut Bugs Trial. Genes on high-quality MAGs (high-quality genes) were used for functional analysis. COG functional annotations for high-quality genes in each sample were obtained using eggNOG. The relative abundance of each COG functional category was calculated as a proportion of the total number of high-quality genes for each sample. Relative abundance data was renormalised for each sample as some genes had multiple functional annotations. Samples with no high-quality genes were omitted. Points are coloured by FMT donors. The grey shaded region represents the 95% confidence interval. FOCUS Trial degrees of freedom = 110; Gut Bugs Trial degrees of freedom = 154. FMT, faecal microbiota transplantation; MAG, metagenome-assembled genome; COG, clusters of orthologous groups.

Podlesny et al^10^ demonstrated that while engraftment was positively affected by the extent of microbiome dissimilarity between pre-treatment recipients relative to a healthy control, this trend was not preserved when comparing the microbiome profiles of pre-treatment recipients to the donor cohort. To test this observation in the FOCUS and Gut Bugs trials, donor and recipient microbial community dissimilarity (*β*-diversity) was calculated using the Bray-Curtis dissimilarity index,^56^ which compares different species present in each sample. When comparing all donors to their respective recipients in the FOCUS Trial, engraftment efficiency was found to be negatively correlated with the mean donor-recipient species Bray-Curtis *β*-diversity value (i.e. more dissimilar species profiles were associated with reduced engraftment efficiency) (Pearson’s correlation, *p* = 2.63 × 10^−04^, *R*² = 0.075) (Figure 5a). However, there was no significant correlation of strain engraftment efficiency with mean donor-recipient species Bray-Curtis *β*-diversity in the Gut Bugs Trial (Pearson’s correlation, *p* = 0.901, *R*² = 1.01 × 10^−04^) (Figure 5c). A similar pattern was observed when assessing the relationship between donor and recipient microbial *β*-diversity and engraftment efficiency using the Jaccard dissimilarity index^57^: engraftment efficiency was negatively correlated with mean donor-recipient species Jaccard *β*-diversity in the FOCUS Trial (Pearson’s correlation, *p* = 1.29 × 10^−04^, *R*² = 0.082) (Supplementary Figure 12a). However, no significant correlation was found for the Gut Bugs Trial (Pearson’s correlation, *p* = 0.064, *R*² = 0.022) (Supplementary Figure 12b).

Given that engraftment efficiency was lower when FOCUS Trial donor-recipient pairings had more distinct species profiles, the influence of donor-recipient pre-FMT functional dissimilarity on engraftment efficiency was investigated. To compute functional *β*-diversity values for donor and recipient samples, metagenome-assembled genome (MAG) assembly was first performed for the FOCUS Trial. An average of 54,948 contigs with a minimum length of 500 bp were assembled per sample (range: 218−137,471). Across all samples, 2,309 MAGs were assembled, of which 362 (15.7%) were high-quality (>90% completeness and <5% contamination). A total of 829,481 genes belonged to high-quality MAGs, comprising 283,563 non-redundant gene clusters, 83.2% of which could be annotated with a COG functional category. MAG assembly statistics for the Gut Bugs Trial have been previously published.^58^

When comparing all donors to their respective recipients in the FOCUS Trial, engraftment efficiency was found to be negatively correlated with the mean donor-recipient functional Bray-Curtis *β*-diversity value (Pearson’s correlation, *p* = 1.87 × 10^−04^, *R*² = 0.120) (Figure 5b). This observation was consistent with the trend observed for species *β*-diversity (Figure 5a), however, the degree of dissimilarity was less when comparing functional profiles. This is likely due to the relatively limited set of possible defined COG functional categories (*n* = 23) present in each sample. The inverse correlation of functional *β*-diversity with engraftment efficiency also revealed a distinct separation of the two most prevalent donors in the FOCUS Trial: D043 (*n* recipients = 29) and D031 (*n* recipients = 26), who had markedly different mean engraftment efficiency across their recipients (15.73% and 4.92%, respectively). No significant correlation between mean donor-recipient functional Bray-Curtis *β*-diversity and engraftment efficiency was observed for the Gut Bugs Trial (Pearson’s correlation, *p* = 0.638, *R*² = 1.44 × 10^−03^) (Figure 5d).

**Figure 6.**
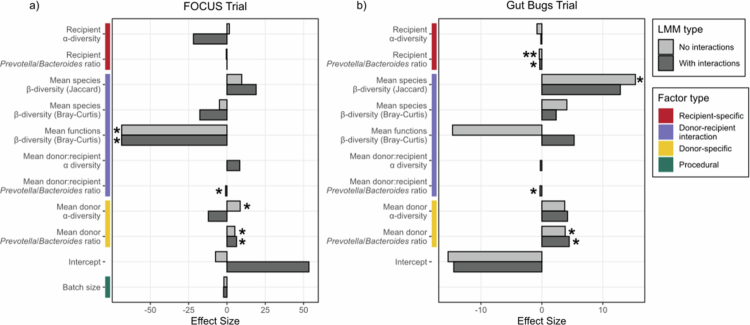
The influence of biological metrics on recipient-specific donor engraftment efficiency differs across FMT trials. Engraftment efficiency was compared for each true donor-recipient pairing using a linear mixed model in (a) the FOCUS Trial and (b) the Gut Bugs Trial. Donor and recipient *α*-diversity (Shannon index), donor and recipient *Prevotella*/*Bacteroides* ratio, species *β*-diversity (Bray-Curtis dissimilarity index and Jaccard dissimilarity index), functional *β*-diversity (Bray-Curtis dissimilarity index) and batch size (FOCUS Trial only) were considered fixed effects. Within-donor and within-recipient variation were considered random effects. For each dataset, the fixed effect estimates are plotted for both the full model (interactions between donor and recipient *α*-diversity, and between donor and recipient *Prevotella*/*Bacteroides* ratio; dark grey bars) and reduced model (no interactions between the fixed effects; light grey bars). Fixed effects are grouped as recipient-specific, donor-recipient interaction, donor-specific, or procedural factors (represented by coloured vertical bars to the left of each plot). FMT, faecal microbiota transplantation; LMM, linear mixed model; *p < 0.05; **p < 0.01.

To compare associations between the microbial diversity metrics and engraftment efficiency, a linear model was fitted to the engraftment efficiency data, with donor and recipient *α*-diversity, species *β*-diversity (Bray-Curtis and Jaccard dissimilarity indices) and functional *β*-diversity (Bray-Curtis dissimilarity index) as fixed effects. The *Prevotella*/*Bacteroides* ratio (i.e. a representation of gut microbial composition enterotypes that have been previously associated with lifestyle factors, including diet^59^^,^^60^ was used as a proxy for community composition. Batch size (i.e. the number of stool donors per recipient) was also included as a fixed effect for the FOCUS Trial, to assess whether the number of donor microbiomes present in the stool transplant influenced the engraftment efficiency of each constituent. However, this was not considered a fixed effect for the Gut Bugs Trial, as each recipient received FMT from exactly four donors. For the FOCUS Trial, the full model with interactions fitted better (ANOVA, *p* = 0.021), while the reduced model with no interactions between fixed effects better fitted the Gut Bugs Trial data (ANOVA, *p* = 0.097). Based on the best fitting model for each trial dataset, there was a significant positive effect of mean donor *Prevotella*/*Bacteroides* ratio (LMM, *b* = 6.28, 95%CI [3.36, 8.84], *p* = 0.023) on engraftment efficiency in the FOCUS Trial. Additionally, there were significant negative effects of the interaction between mean donor *Prevotella*/*Bacteroides* ratio and recipient *Prevotella*/*Bacteroides* ratio (LMM, *b* =–0.899, 95%CI [–1.69, –0.243], *p* = 0.017) and mean donor-recipient functional *β*-diversity (Bray-Curtis dissimilarity index) (LMM, *b* = –68.7, 95%CI [–121, –21.3], *p* = 0.034) on engraftment efficiency (Figure 6a). A subsequent linear model with the mean difference in donor and recipient individual COG functional category normalised relative abundances did not identify any significant effects. This finding suggests that the impact of functional *β*-diversity on engraftment efficiency is not driven by a single function. Instead, it is likely due to the complementarity of microbiome functions for each donor-recipient pairing. Conversely, for the Gut Bugs Trial, there was a significant positive effect of mean donor-recipient species *β*-diversity (Jaccard dissimilarity index) (LMM, *b* = 15.3, 95%CI [2.09, 29.1], *p* = 0.031) and mean donor *Prevotella*/*Bacteroides* ratio (LMM, *b* = 3.83, 95%CI [1.47, 6.17], *p* = 0.036) on engraftment efficiency. There was an additional significant negative effect of recipient *Prevotella*/*Bacteroides* ratio (LMM, *b* =–0.424, 95%CI [–0.680, –0.159], *p* = 3.77 × 10^−03^) on engraftment efficiency (Figure 6b). These results suggest that the factors influencing engraftment efficiency share similarities and differences across FMT trial cohorts, and that while the interaction between donor and recipient microbiomes were important factors in both the FOCUS and Gut Bugs cohorts, the specific interaction dynamics were unique to each group.

Finally, it is possible that the species and functional profiles of different samples may have been impacted by sequencing depth, as species with lower relative abundance may not have met the threshold required for detection. This impact may be more relevant for the FOCUS Trial which had a sequencing depth of approximately 4.4 million microbial reads per sample,^11^compared with an average of 45.7 million microbial reads per sample in the Gut Bugs Trial.^13^ To test this, a principal coordinate analysis was performed on functional and species Bray-Curtis and Jaccard *β*-diversity in each trial. There was a significant impact of sample read count on species Bray-Curtis *β*-diversity (PERMANOVA, *p* < 0.001, *R*² = 0.065) (Supplementary Figure 13a) and functional Bray-Curtis *β*-diversity (PERMANOVA, *p* = 0.019, *R*² = 0.066) (Supplementary Figure 13b) in the FOCUS Trial, suggesting that the significant effect of these factors on engraftment efficiency may be an artefact of low sequencing depth. Functional Bray-Curtis *β*-diversity of samples in the Gut Bugs Trial, which did not have a significant effect on recipient-specific donor engraftment efficiency in the above linear mixed model, was not significantly impacted by sample read count (PERMANOVA, *p* = 0.333, *R*² = 0.011) (Supplementary Figure 13d). However, there was a significant impact of sample read count on species Bray-Curtis *β*-diversity in the Gut Bugs Trial (PERMANOVA, *p* = 0.049, *R*² = 0.016) (Supplementary Figure 13c), suggesting that this effect can occur even at greater sequencing depths. Similarly, the impact of sample read count on Jaccard species *β*-diversity in the FOCUS Trial was significant (PERMANOVA, *p* < 0.001, *R*² = 0.096) (Supplementary Figure 14a). However, no significant impact was observed in the Gut Bugs Trial (PERMANOVA, *p* = 0.123, *R*² = 0.013) (Supplementary Figure 14b).

## Discussion

### Influences on donor strain engraftment

This study aimed to test the feasibility of donor strain matching as an approach to predict donor-recipient pairings from an FMT trial dataset. A range of normalised DNA distance thresholds, which were used to identify genetic strain matches present in recipients and their respective donor samples, were assessed by F1 score to determine the optimal threshold with the best precision and recall of donor-recipient pairings. A strain engraftment analysis was performed using this optimal threshold, which revealed a range in the ability to recall the different FMT donors used in the FOCUS Trial for UC (0−83%).^2^^,^^11^ As anticipated, donor recall correlated with the proportion of donor strains engrafted across their recipient cohort (i.e. engraftment efficiency). A second, validation analysis was performed on data from Gut Bugs Trial for obesity,^3^^,^^12^^,^^13^ where donor recall ranged from 29−100%. The increase in donor recall for the latter study reflects the impact of sequencing depth on microbial strain detection. The prediction of FMT donor-recipient pairings through strain engraftment analysis is a useful tool in cases where donor-recipient pairings are unable to be obtained for planned analyses, or as a validation of the strain matching approach that is often used to track donor strains within the recipient microbiome after treatment.

Donor engraftment after FMT is an important consideration in trial design, due to its association with treatment success.^23^^,^^24^ Previous meta-analyses of FMT trial datasets^10^^,^^23^^,^^28^ have highlighted a number of factors that may contribute to variability in donor engraftment. These factors range in nature from trial design (e.g. FMT pre-treatment, number of FMT donors, FMT treatment route, number of FMT doses), to the condition of the donor microbiome (*α*-diversity), the condition of the recipient microbiome (*α*-diversity and level of dysbiosis), and the complementarity of the donor and recipient microbial communities. Analyses of FMT data, for example those that track the appearance and persistence of novel donor-matching strains in the recipient microbiome after FMT treatment, are further influenced by trial design. Tools such as StrainPhlAn3,^38^ which can be used to profile the strain composition of metagenomes, require a minimum genome coverage to produce a consensus sequence for the dominant species strain in a metagenome. Therefore, lower sequencing depths, as well as low abundance of the microbial strains themselves within samples, will limit their identification. In the FOCUS Trial, metagenomic sequencing was performed to an average depth of 4.4 (±2.0) million microbial reads per sample (post-QC), which may have contributed to the limited profiling of strains across the selected samples (*n* distinct engrafted donor strain species = 19).^11^ For comparison, the Gut Bugs Trial had an average of 45.7 (±6.1) million microbial reads per sample (post-QC),^13^and strain profiling identified 57 distinct engrafted donor strain species. The initial blinded strain engraftment analysis of FOCUS Trial data further highlights the likely connection between sequencing depth, donor strain profiling, and the prediction of FMT donor-recipient pairings. This model achieved a precision of 0.805 and a recall of 0.405, suggesting that while few pairings were able to be recalled, the proportion of predictions that were able to be made based on donor-matching strains were largely correct. In a similar manner, assemblies using longer sequencing read lengths will enable microbial identification with greater precision.^61^ The introduction of bias is possible at many trial stages, from sample collection to data processing,^62^ leading to the possible missed detection of less common species, or the variability in their detection across samples. Indeed, a recent study has highlighted the importance of sample collection timing in microbiome analyses.^63^ Nonetheless, the consideration of these known factors reveals a persisting element of stochasticity in donor strain engraftment,^28^ suggesting there may be further unknown factors of importance.

### Variability of donor strain engraftment

After identifying disparities in the engraftment efficiency of individual FMT donors from the FOCUS Trial, a number of possible underlying factors were considered, from those previously highlighted through meta-analyses.^10^^,^^23^^,^^28^ Notably, participants in the FOCUS Trial were not pre-treated with antibiotics, contrasting this analysis with previous studies that have evaluated the effect of antibiotic pre-treatment on FMT outcomes in people with UC or other inflammatory bowel diseases.^64–67^ The *α*-diversity of microbes within the donor stool transplant could be viewed as a proxy for the complement of microbial strains with the potential to engraft in the recipient gut. In contrast, the microbial *α*-diversity present in the recipient gut at the time of treatment could be viewed as a proxy for the ecological niches already occupied or the potential competition for transplanted donor strains, as well as the existing network of functional capabilities. In the initial blinded analysis of the FOCUS Trial samples, strains originating from donor D043 were predicted in 75% (24/32) of recipients; the highest of any donor. However, it was also noted that relative to the donor pool, samples from D043 did not exhibit high *α*-diversity. This trend was further observed in the unblinded analysis, where donor *α*-diversity was not significantly correlated with mean engraftment efficiency. However, a greater proportion of donors with mean *α*-diversity above 2.5 exhibited a non-zero level of engraftment efficiency in their recipients, suggesting there may be a minimal donor *α*-diversity threshold required for effective engraftment. A mean *α*-diversity threshold of 2.5 was exceeded by all donors in the Gut Bugs Trial. The best performing donor in the Gut Bugs Trial, DF16, did exhibit the highest mean *α*-diversity. However, overall, there was also no significant correlation between mean donor *α*-diversity and engraftment efficiency in the analysis of the Gut Bugs Trial data. Recipient *α*-diversity in the FOCUS Trial was significantly reduced compared with the donors, which is consistent with previous findings where UC has been associated with a reduction in gut microbial diversity.^15^ Obesity has also previously been associated with a reduction in diversity,^19^ however, a significant difference was not observed in this analysis. This may have been due to reduced samples sizes, with only nine donors used throughout the Gut Bugs Trial. However, in both trials, there were cases where the recipient *α*-diversity exceeded that of the donor. Therefore, it is conceivable that it is not just the donor species richness that is an important factor in FMT engraftment, but also the complementarity of the donor species with those that persist in the recipient’s gut.

The value of microbiome complementarity was observed through significant correlations between engraftment efficiency and donor-recipient functional (COG) Bray-Curtis *β*-diversity in the FOCUS Trial. However, it was not the difference but rather the relative similarity of functional profiles that was associated with increased engraftment efficiency, highlighting the importance of microbial complementarity for the retention of novel species within the recipient microbiome. Intuitively, this correlation must not be entirely linear, as it is not expected that donor-recipient pairings with entirely similar profiles would exhibit the highest level of engraftment efficiency. UC has been previously associated with the altered composition of the gut microbiome,^16^therefore the FOCUS Trial recipient species profiles would be expected to differ from that of the healthy donors. Moreover, if donor and recipient profiles were to match exactly, all donor species would be lacking available niches within the recipient gut. Obesity has also previously been associated with perturbations of the gut microbiota.^68^ In the analysis of the Gut Bugs Trial data there was no significant correlation initially observed between engraftment efficiency and donor-recipient species- and functional *β*-diversity in the Gut Bugs Trial data. However, when other factors were taken into account, there was a significant effect of species Jaccard *β*-diversity on engraftment efficiency, albeit a positive effect (i.e. more dissimilar species profiles were associated with improved engraftment efficiency). Taken together, these results may suggest a bimodal effect of donor-recipient *β*-diversity on engraftment efficiency, where both increasing and decreasing microbial species or functional complementarity can facilitate engraftment (to an extent), perhaps reflecting specific interactive dynamics between the incoming and endogenous microbial populations in the recipient’s gut.^69^ Nonetheless, the significant effects of *β*-diversity on engraftment efficiency in both trials may have been influenced by sequencing depth. This influence was observed to be more significant in the FOCUS Trial, as at a lower average sequencing depth it is expected that fluctuations in read count would have a greater impact on the ability to detect species presence. It is also possible that the disparate impacts on engraftment efficiency in each trial may be a technical artefact of protocol differences, such as FMT administration route, between the FOCUS and Gut Bugs trials. Consequently, it is pertinent that more FMT trials with greater sequencing depth, and ideally high numbers of participants, are performed on a range of health conditions, in order to better understand the different factors influencing engraftment efficiency and ultimately improve FMT efficacy. In particular, the specific taxa associated with FMT response should be identified for each health condition, and the factors influencing their engraftment explored.

Previous meta-analyses investigating the factors contributing to engraftment after FMT have focussed on identifying general factors across multiple study cohorts with different gut-related health conditions.^10^^,^^23^^,^^28^ These provide important information for the design of future FMT trials to achieve modulation of the recipient microbiome. However, different health conditions (including those that are suitable targets for FMT intervention) are associated with specific gut microbial markers.^54^ Therefore, we hypothesised that different factors will promote engraftment efficiency in recipients with different health conditions. Indeed, the differing factors found to influence strain engraftment in the FOCUS and Gut Bugs trials in this study suggest a need to establish methodologies that are specific to each condition being treated. It is also possible that there may be sub-groups within an FMT cohort where engraftment is influenced by different biological metrics, although further analyses are required to investigate such associations. Ultimately, we suggest that the true potential of FMT will be realised through the lens of personalised medicine, where recipients are screened and paired with one or multiple donors that, based on their condition, will have the best chance to engraft beneficial species to modulate their microbiome and ameliorate the condition for which they are being treated.

## Supplementary Material

Supplementary Material supplementary_fig_2.png

Supplementary Material supplementary_fig_6.png

Supplementary Material supplementary_fig_4.png

Supplementary Material supplementary_fig_8.png

Supplementary Material supplementary_fig_11.png

Supplementary Material supplementary_fig_9.png

Supplementary Material supplementary_fig_12.png

Supplementary Material supplementary_fig_5.png

Supplementary Material supplementary_fig_10.png

Supplementary Material supplementary_fig_13.png

Supplementary Material supplementary_fig_14.png

Supplementary Material supplementary_fig_1.png

Supplementary Material supplementary_fig_7.png

Supplementary Material supplementary_fig_3.png

Supplementary Material Supplementary Material.

## Data Availability

The Gut Bugs Trial and FOCUS Trial metagenomic datasets analysed during the current study are publicly available in the NCBI Sequence Read Archive repository (BioProject PRJNA637785) and European Nucleotide Archive (accession number PRJEB26357), respectively. Additional data files are available on request. Data processing R scripts are available at https://github.com/annabehling/strain_engraftment/. Supplementary files can be found at doi.org/10.17608/k6.auckland.29095370. A draft manuscript of the blinded analysis results written prior to obtaining the true FMT donor-recipient pairings on 15/05/2024 can be found at doi.org/10.17608/k6.auckland.25823902 (the data will be made publicly available upon submission of the manuscript).
