# Supplementary figures and images for: Cohort-specific determinants of donor strain engraftment following multi-donor faecal microbiota transplantation in two randomised clinical trials
Source: Gut Microbes. 2025 Dec 11;17(1):2597628. doi: 10.1080/19490976.2025.2597628 (PMC12710892; doi:10.1080/19490976.2025.2597628)

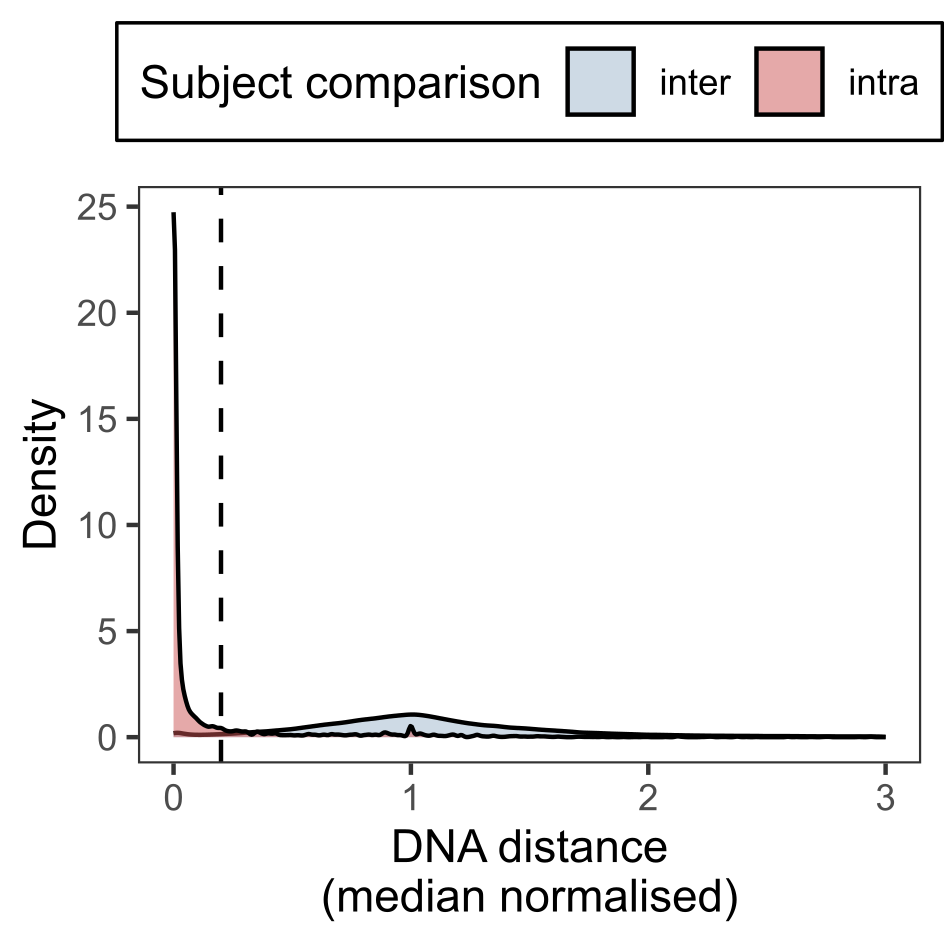

Supplement: Supplementary Material — supplementary_fig_2.png [file KGMI_A_2597628_SM6739.png]

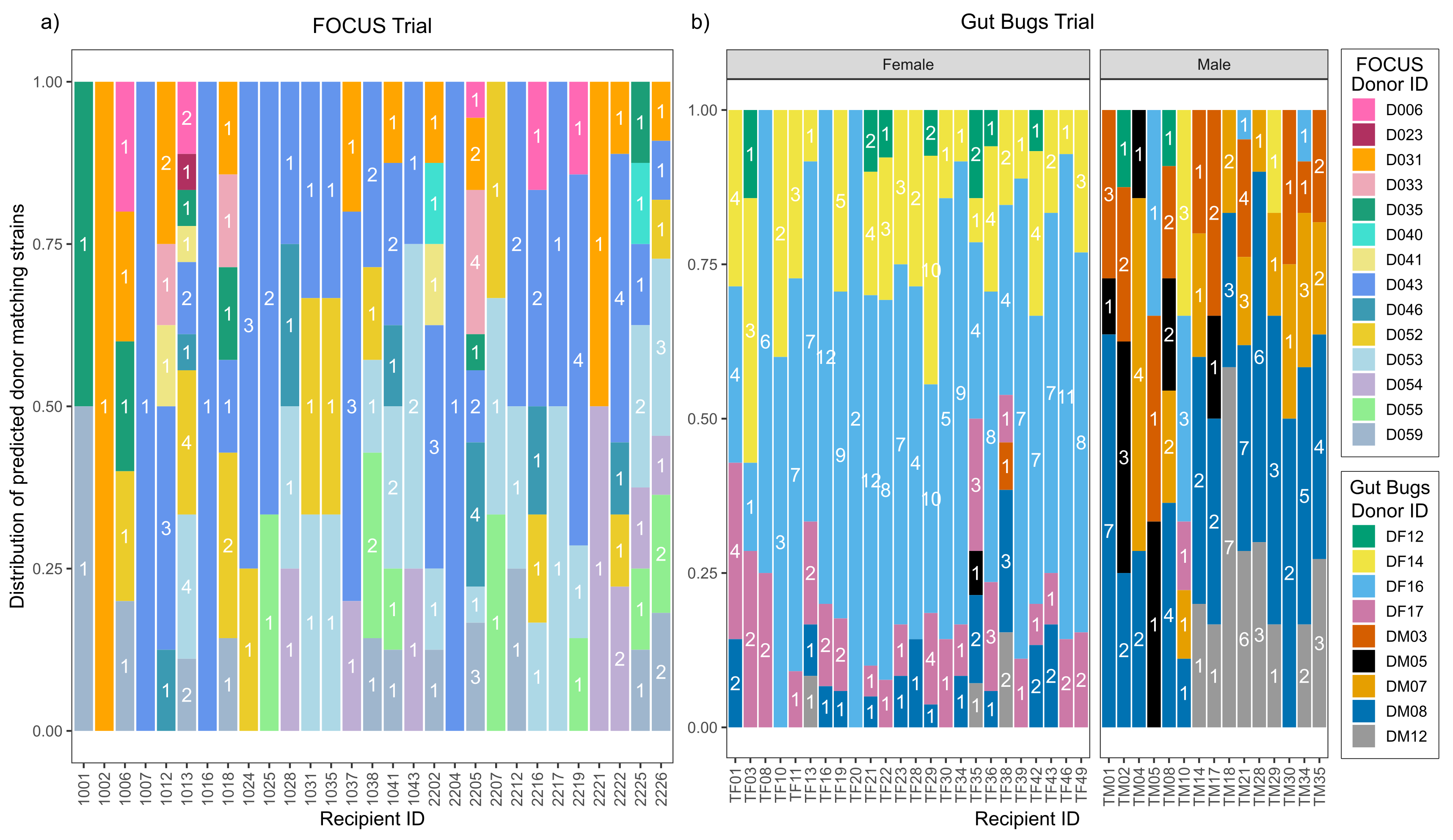

Supplement: Supplementary Material — supplementary_fig_6.png [file KGMI_A_2597628_SM6740.png]

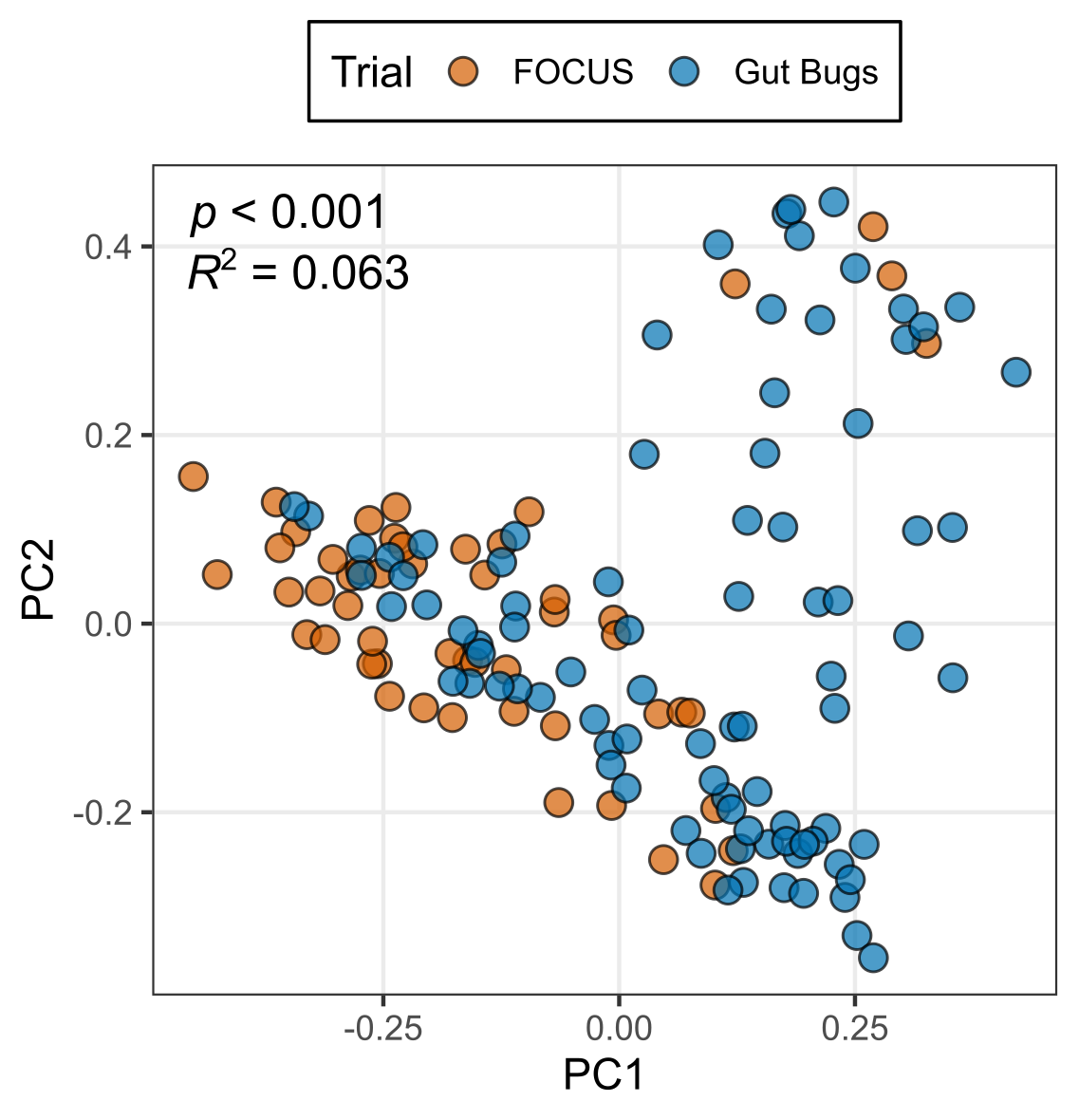

Supplement: Supplementary Material — supplementary_fig_4.png [file KGMI_A_2597628_SM6741.png]

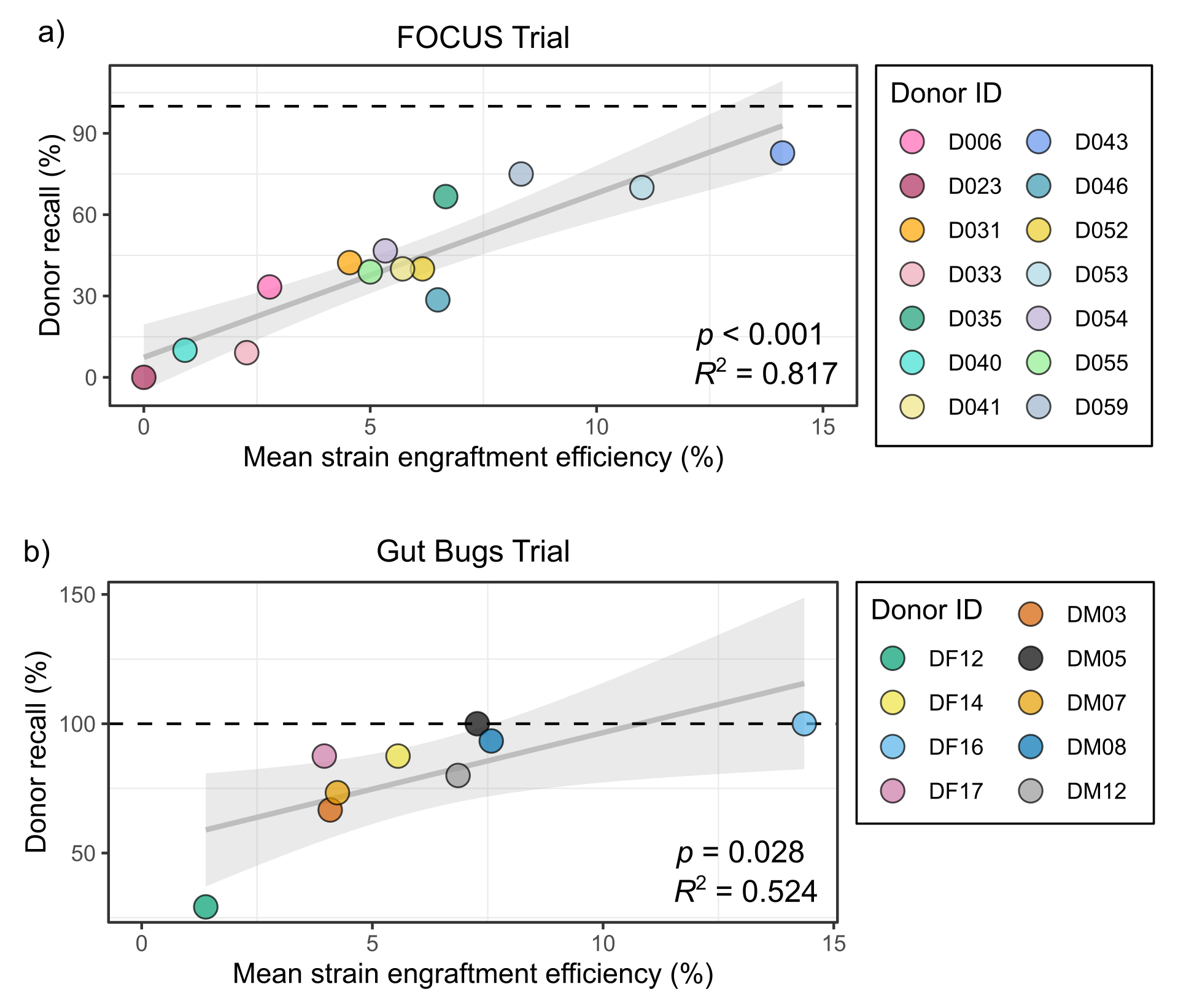

Supplement: Supplementary Material — supplementary_fig_8.png [file KGMI_A_2597628_SM6742.png]

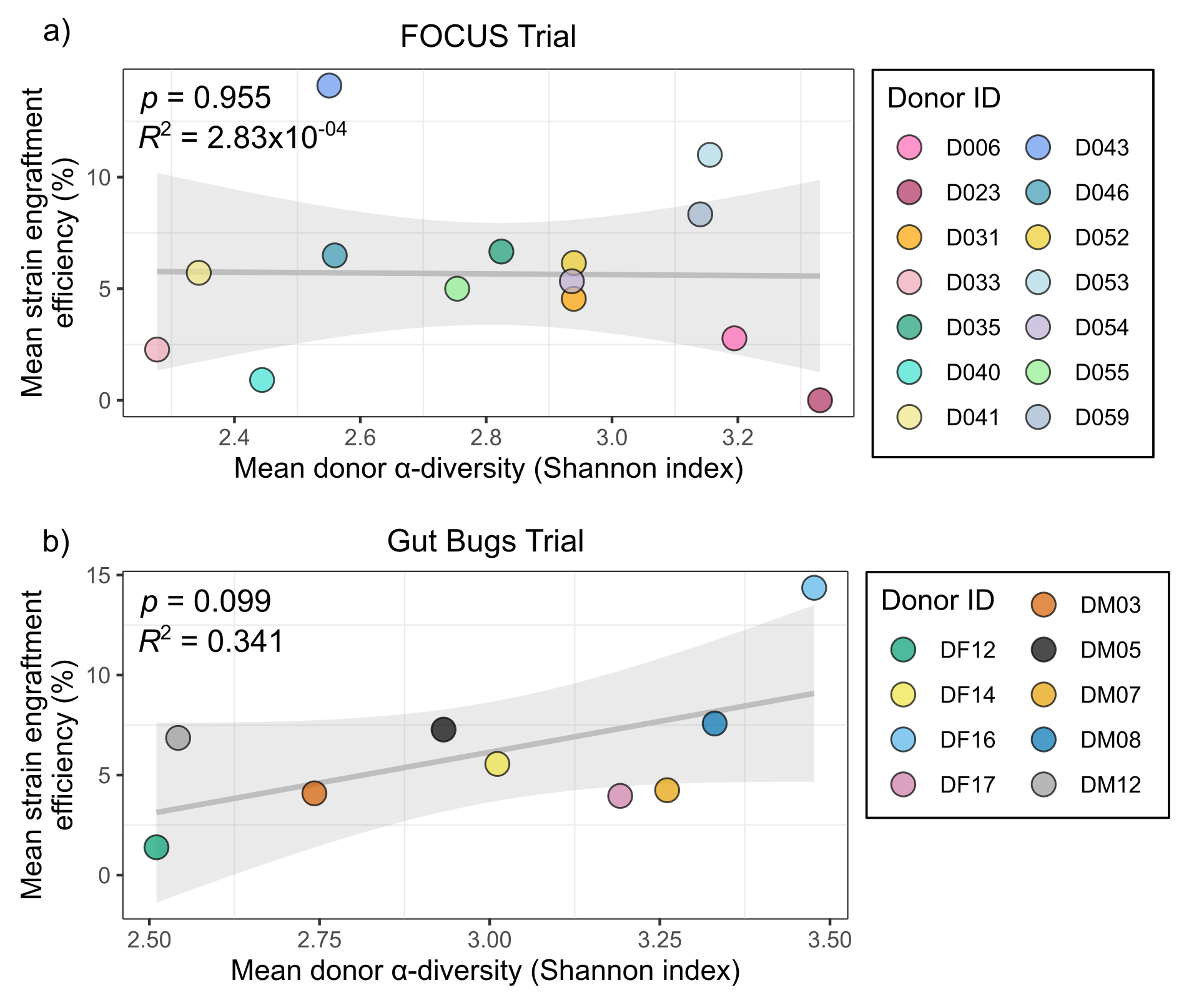

Supplement: Supplementary Material — supplementary_fig_11.png [file KGMI_A_2597628_SM6743.png]

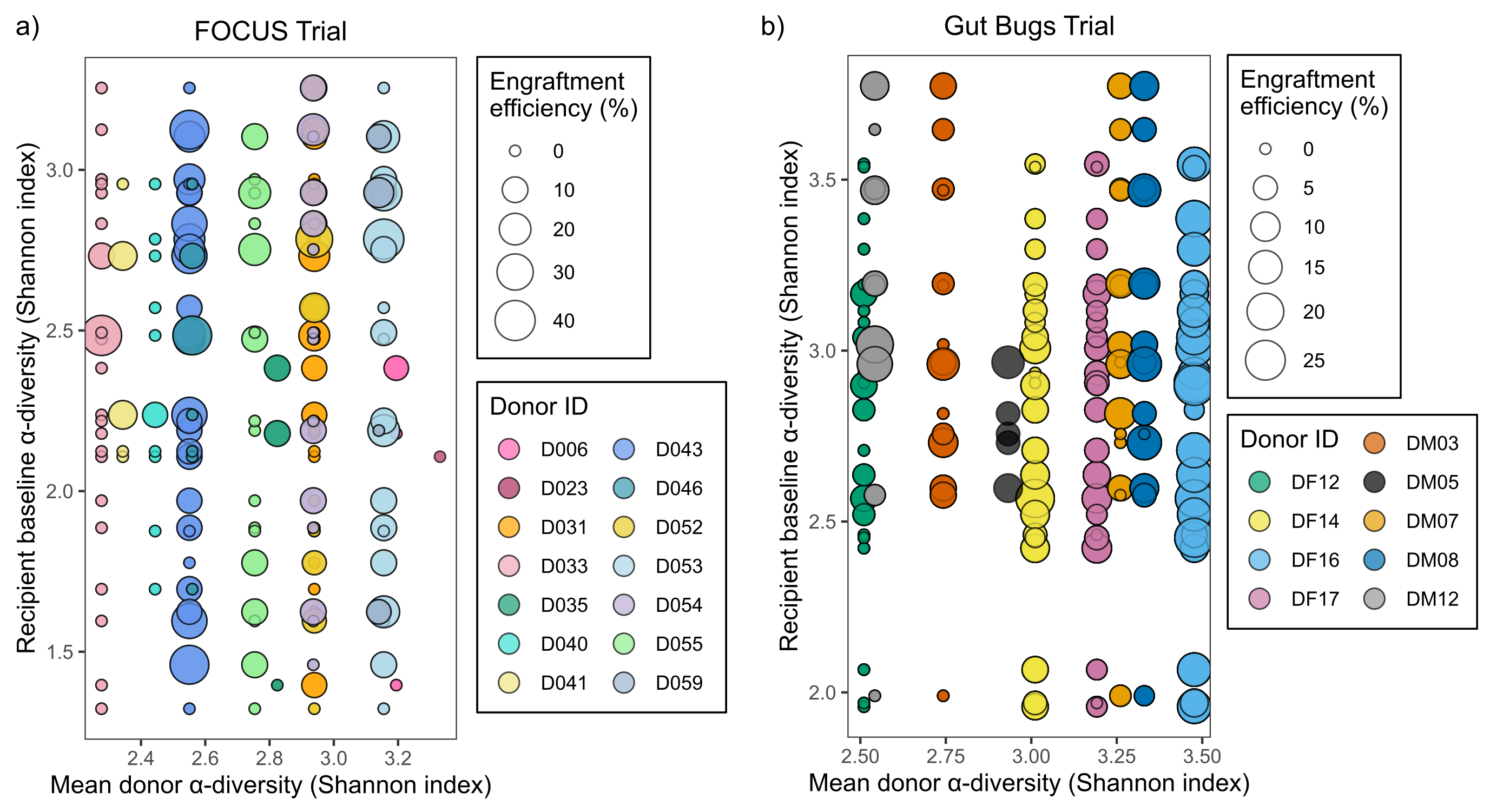

Supplement: Supplementary Material — supplementary_fig_9.png [file KGMI_A_2597628_SM6744.png]

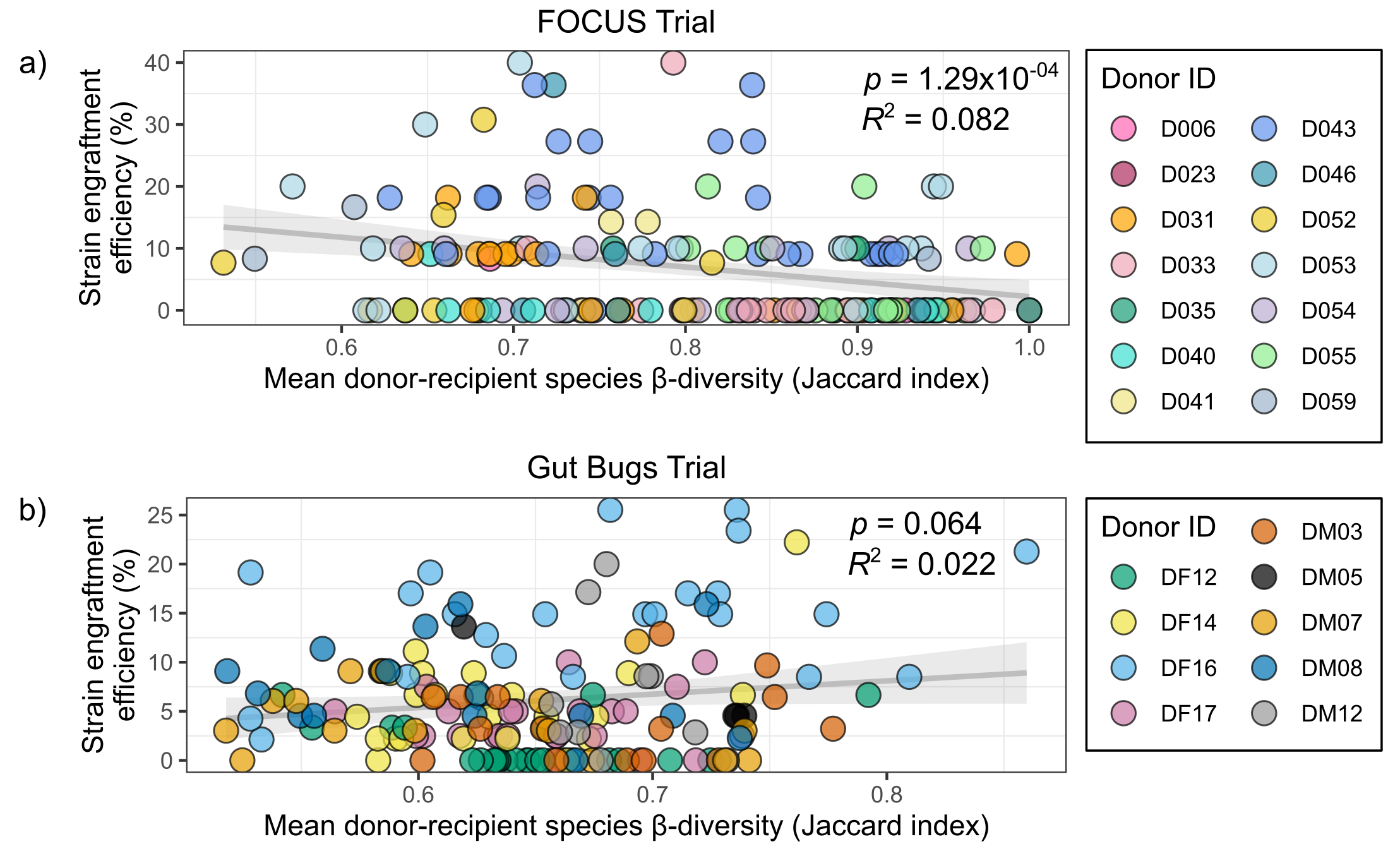

Supplement: Supplementary Material — supplementary_fig_12.png [file KGMI_A_2597628_SM6745.png]

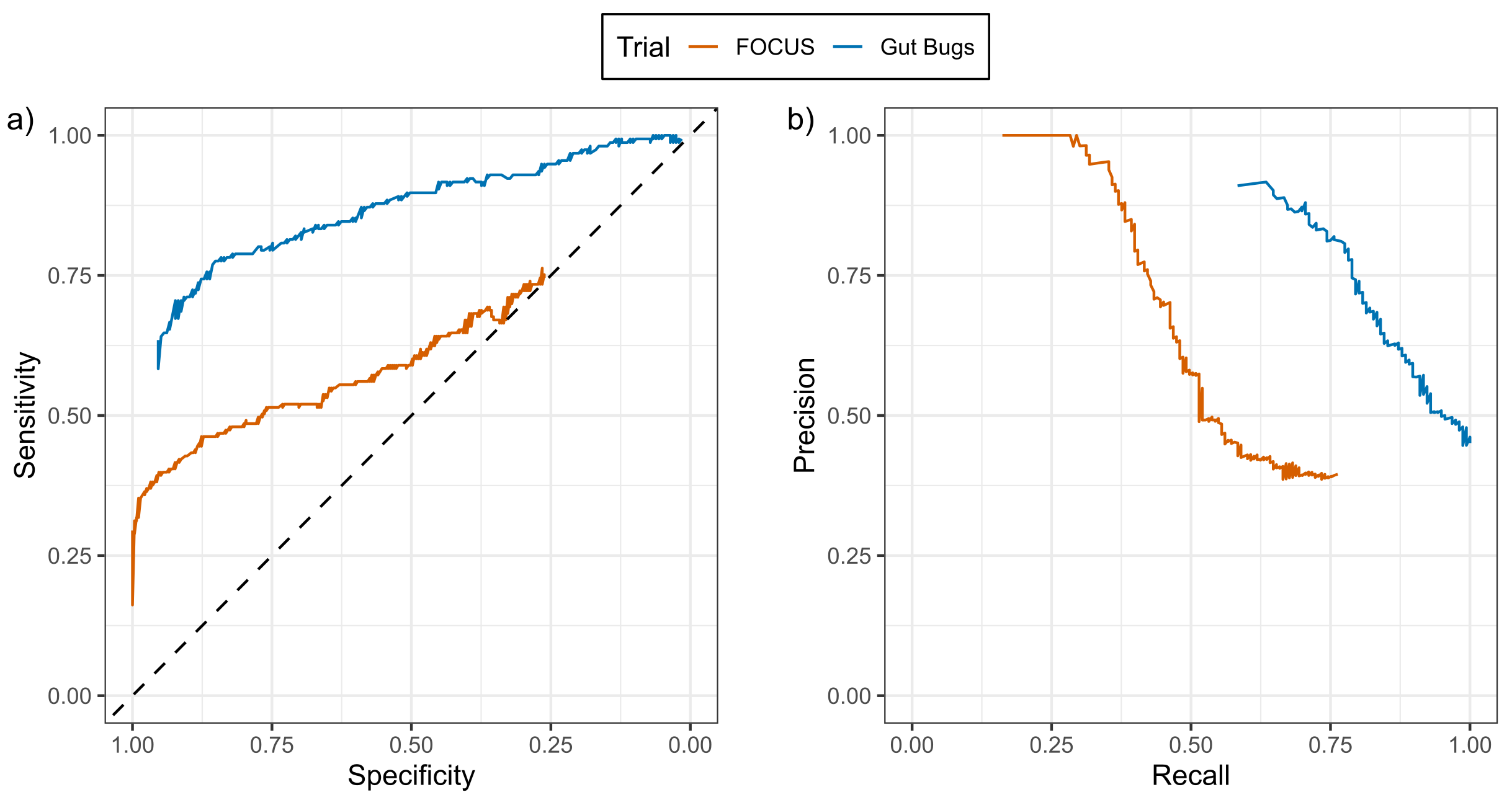

Supplement: Supplementary Material — supplementary_fig_5.png [file KGMI_A_2597628_SM6746.png]

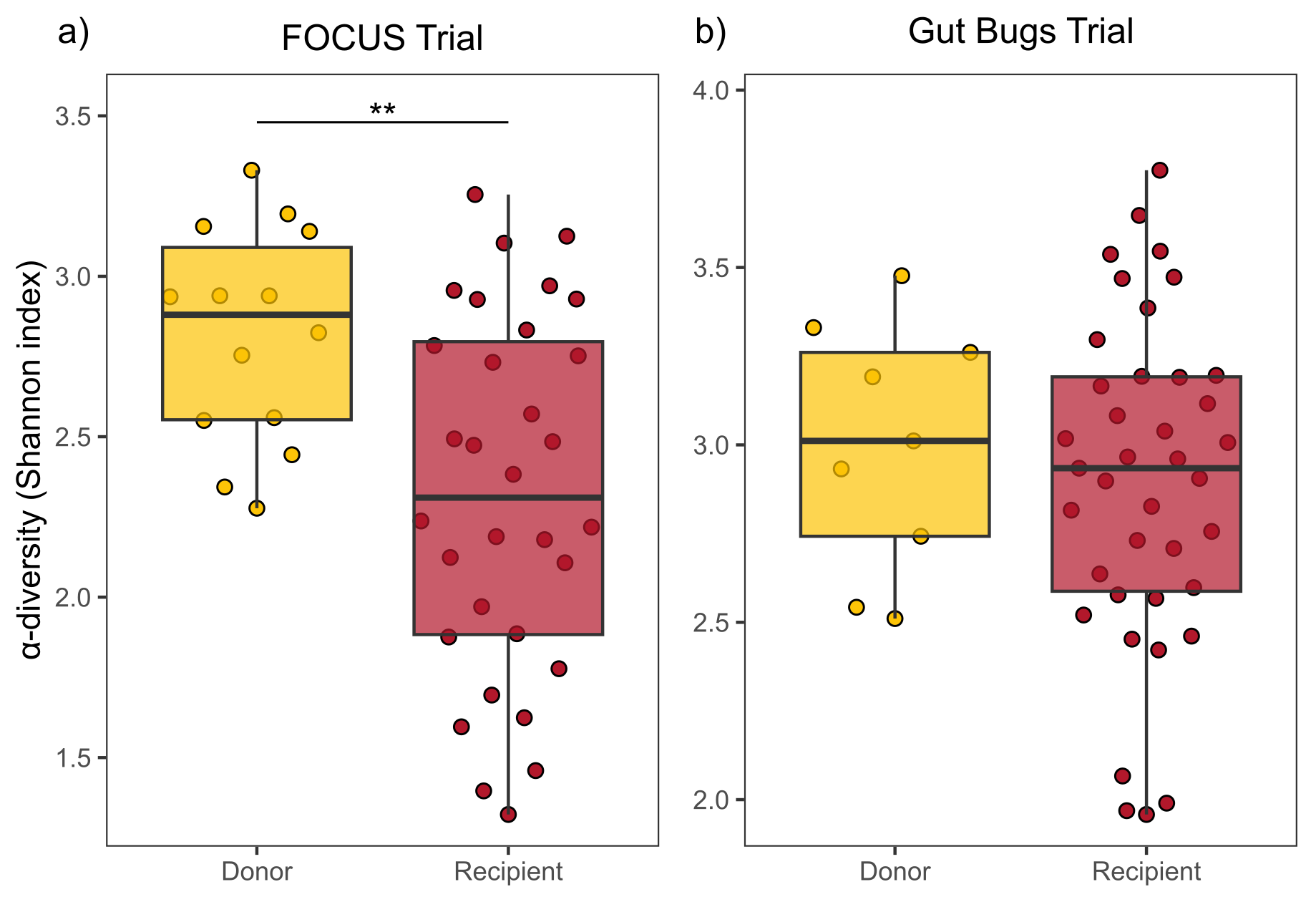

Supplement: Supplementary Material — supplementary_fig_10.png [file KGMI_A_2597628_SM6747.png]

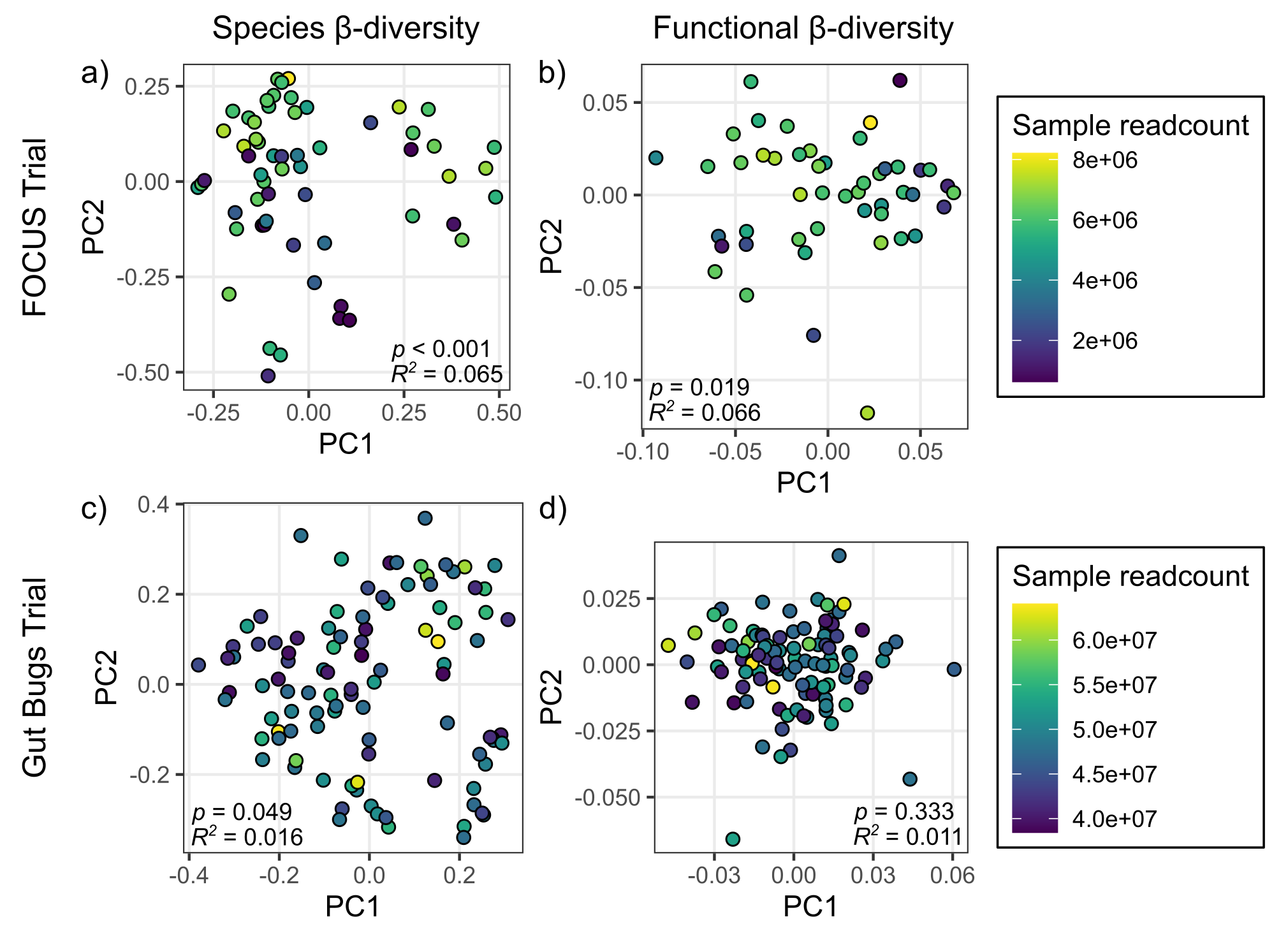

Supplement: Supplementary Material — supplementary_fig_13.png [file KGMI_A_2597628_SM6748.png]

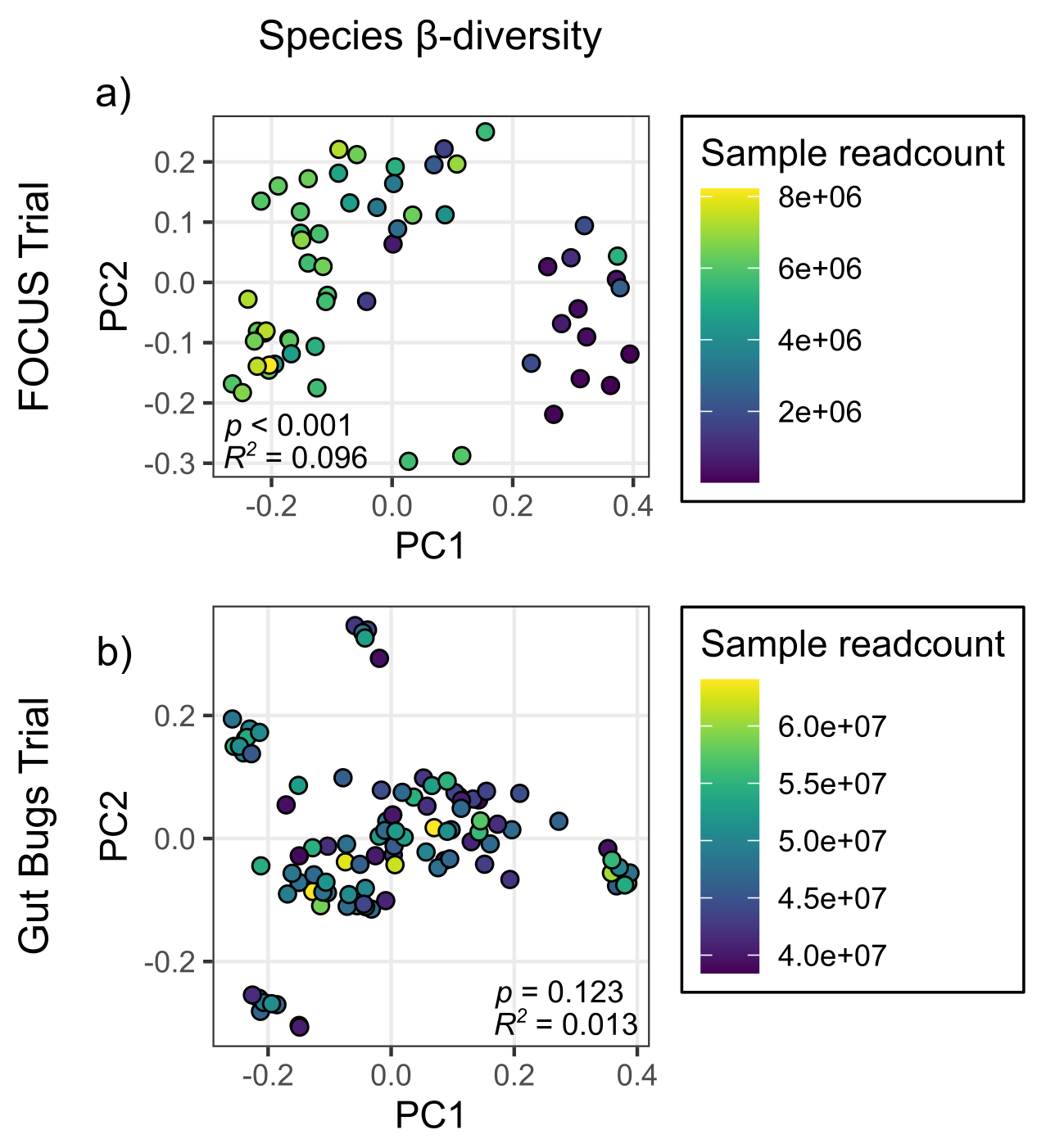

Supplement: Supplementary Material — supplementary_fig_14.png [file KGMI_A_2597628_SM6749.png]

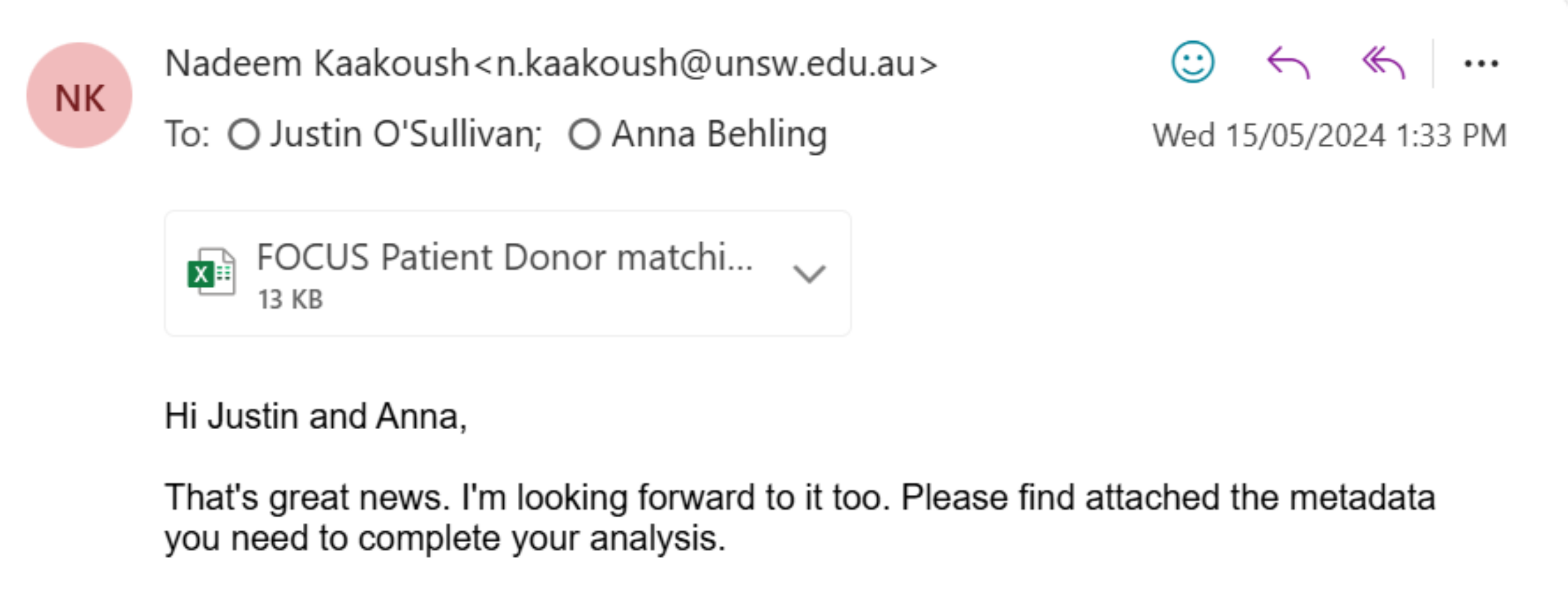

Supplement: Supplementary Material — supplementary_fig_1.png [file KGMI_A_2597628_SM6750.png]

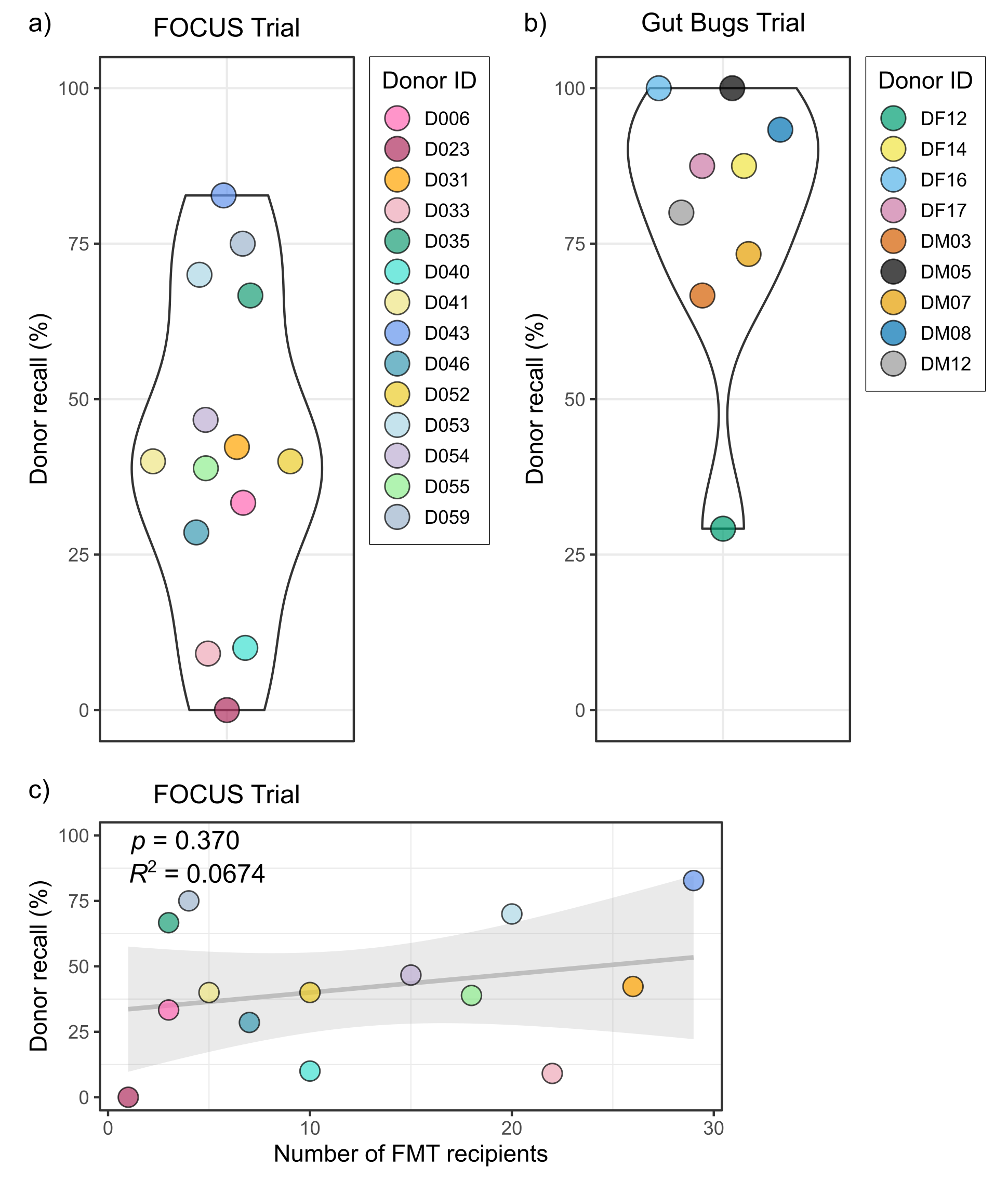

Supplement: Supplementary Material — supplementary_fig_7.png [file KGMI_A_2597628_SM6751.png]

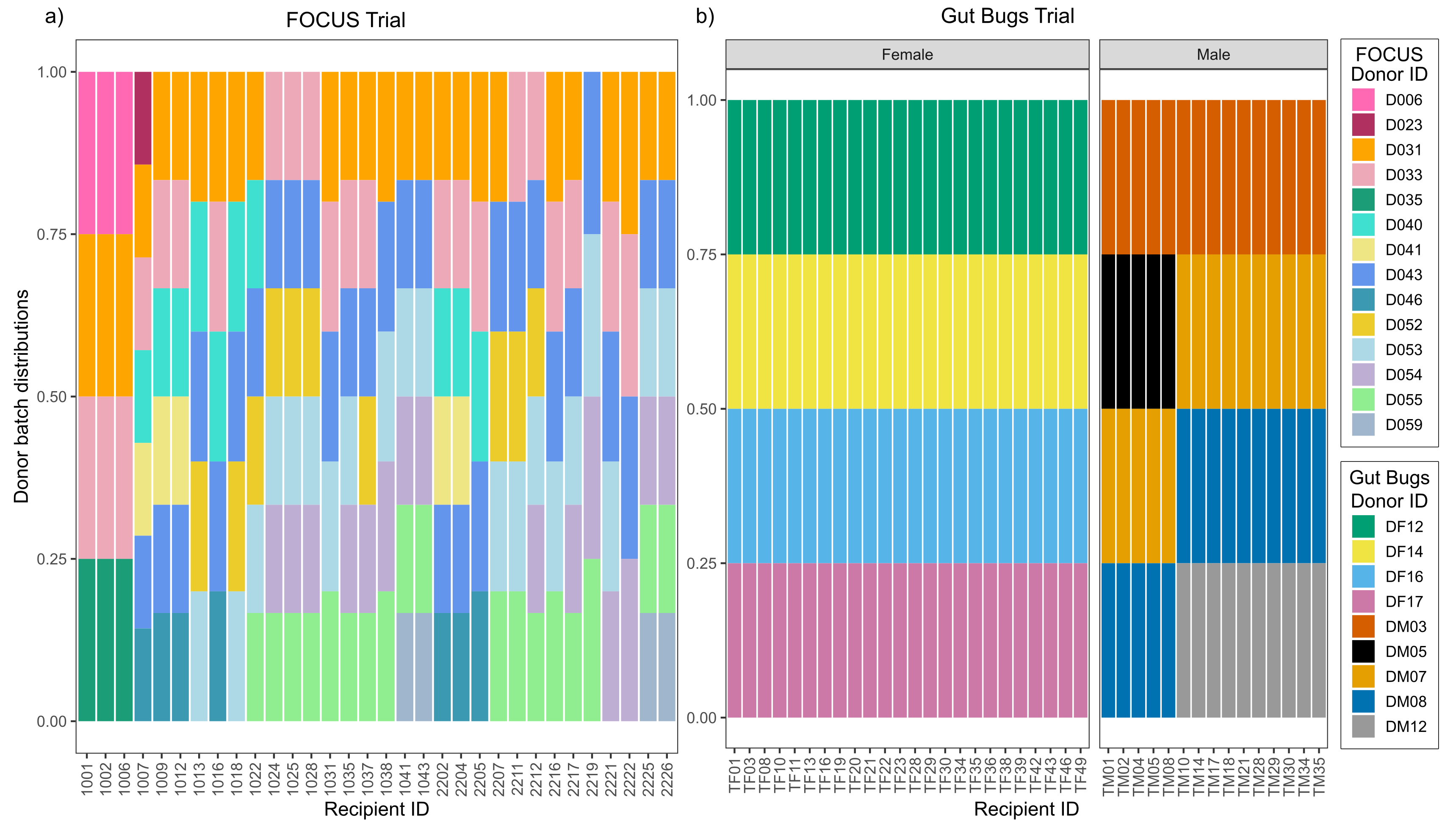

Supplement: Supplementary Material — supplementary_fig_3.png [file KGMI_A_2597628_SM6752.png]
