# Supplementary material for: Cohort-specific determinants of donor strain engraftment following multi-donor faecal microbiota transplantation in two randomised clinical trials
Source: Gut Microbes. 2025 Dec 11;17(1):2597628. doi: 10.1080/19490976.2025.2597628 (PMC12710892; doi:10.1080/19490976.2025.2597628)
Supplement: Supplementary Material [file KGMI_A_2597628_SM8252.docx]

**Supplementary figures**

**
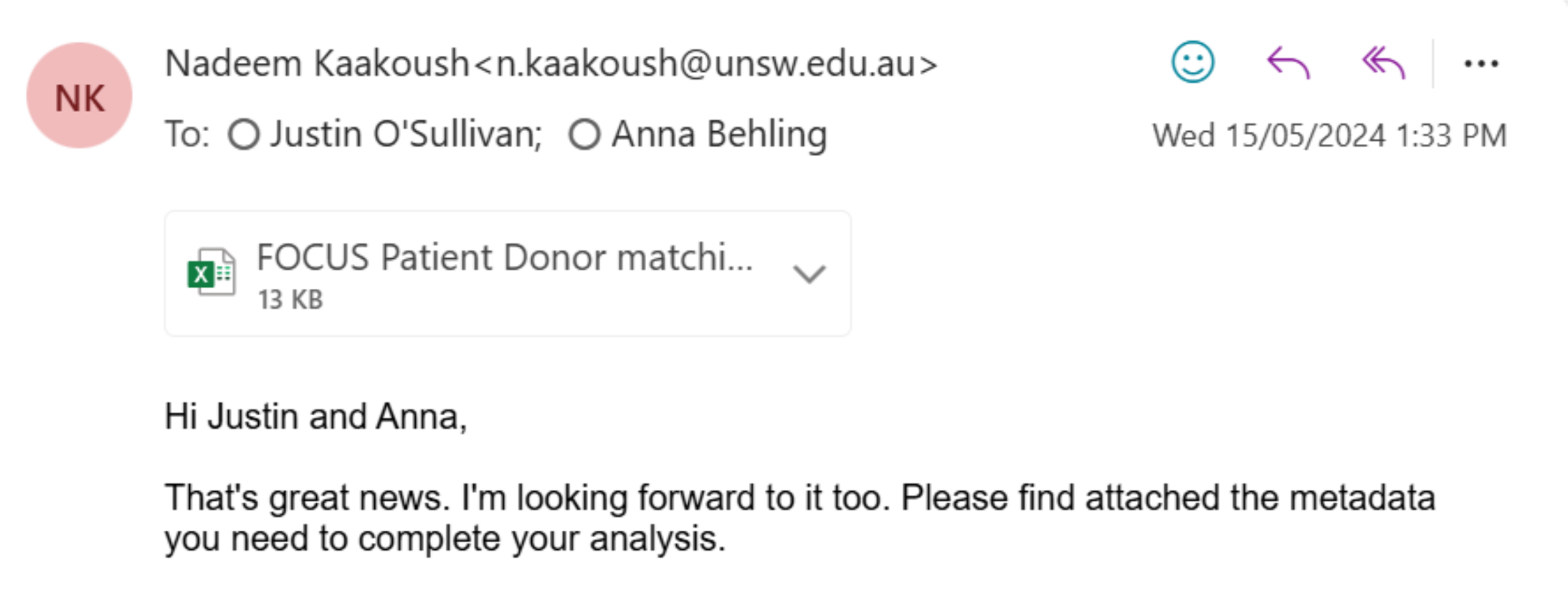
**

**Supplementary Figure 1. Email correspondence containing metadata for the FOCUS Trial.**


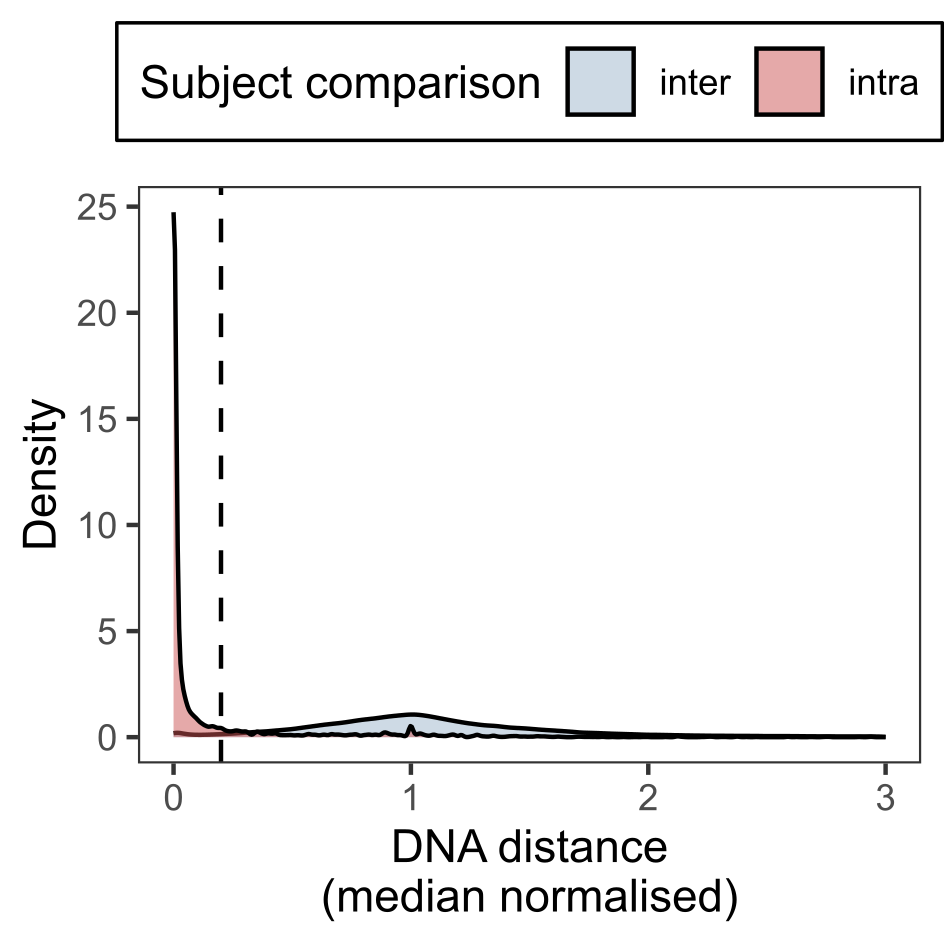


**Supplementary Figure 2. Strain matching profile for the Gut Bugs Trial.** The density of DNA distance (median normalised) values for strains from inter- (grey) and intra-subject (pink) pairwise comparisons is plotted. Donor batch samples were excluded from pairwise comparisons due to their ambiguity as intra- or inter-subject comparisons. Total pairwise comparisons considered = 641,108. Pairwise comparisons > 3 were not plotted due to their low density. Strains with ≤ 0.2 normalised DNA distance (dashed line) were considered a strain match in a previous analysis [13].


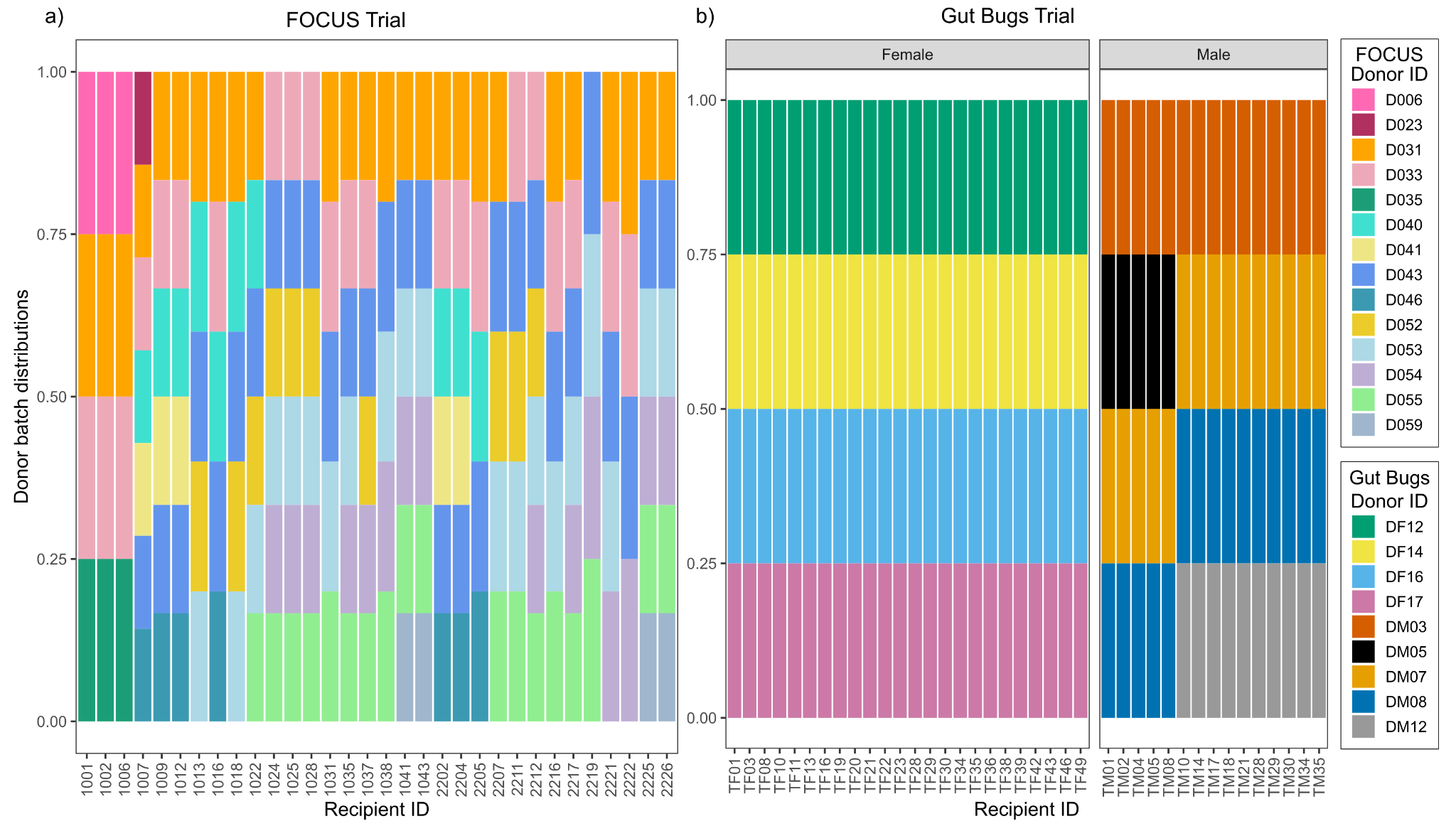


**Supplementary Figure 3. True FMT donor-recipient pairings used in each trial.** (a): In the FOCUS Trial, the 32 FMT recipients (count at week 8) each received a range of 4-7 donor microbiomes in the FMT treatment. On average, each donor was used in 12 recipient batches (range: 1 (D023) - 29 (D043)). (b): In the Gut Bugs Trial, the 39 FMT recipients (count at week 6) each received 4 sex-matched donor microbiomes in the FMT treatment. Male donor DM05 was replaced by DM12 during the trial. Bars are coloured by FMT donors. Bar height corresponds to the donor contribution in each batch. FMT, faecal microbiota transplantation.


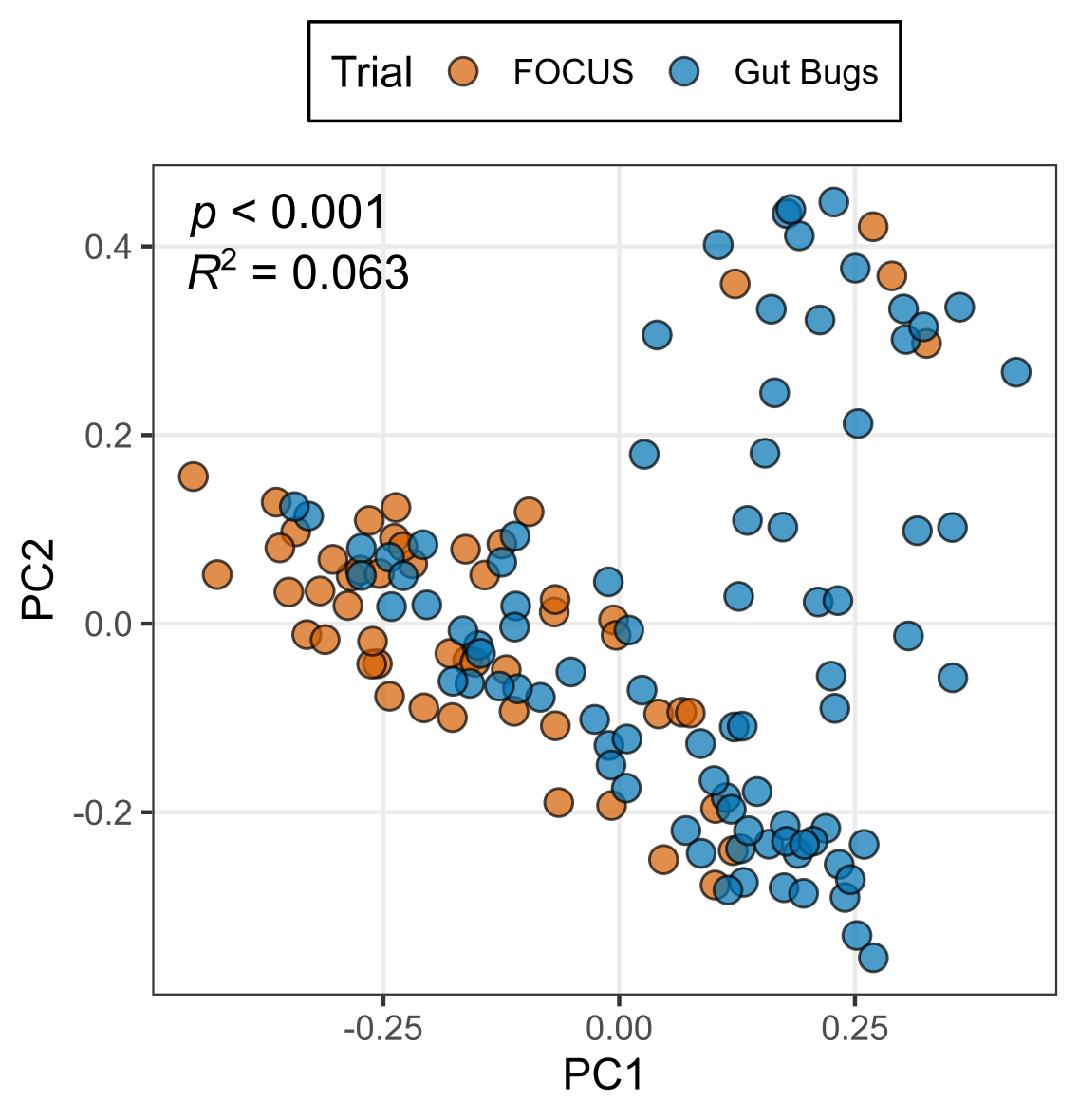


**Supplementary Figure 4. The gut microbiota profiles in ulcerative colitis and obesity were significantly different.** Classical (metric) multidimensional scaling of species relative abundance data for all recipient baseline samples (FMT and placebo) in the FOCUS (orange) and Gut Bugs (blue) trials, using the Bray-Curtis dissimilarity index. Species profiles were obtained from MetaPhlAn3. Each point represents a sample. Coordinates are equal. FMT, faecal microbiota transplantation; PC1, principal coordinate 1; PC2, principal coordinate 2.


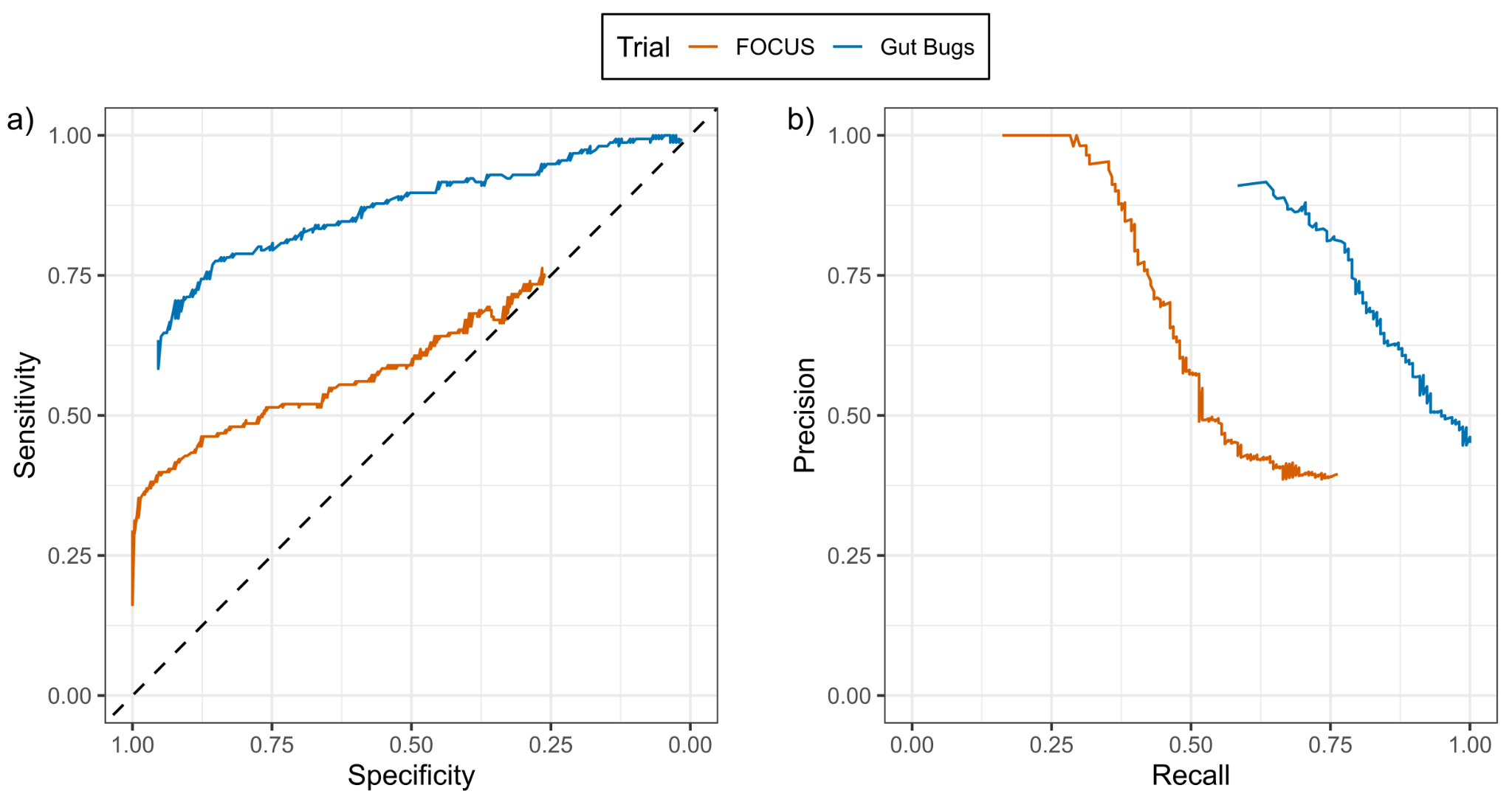


**Supplementary Figure 5. Donor strain matching to predict FMT donor-recipient pairings was more effective on the Gut Bugs Trial dataset.** (a) Sensitivity-specificity and (b) precision-recall curves for donor strain matching using normalised DNA distance thresholds 0.001-3 for the FOCUS (orange) and Gut Bugs (blue) trials. FMT, faecal microbiota transplantation.


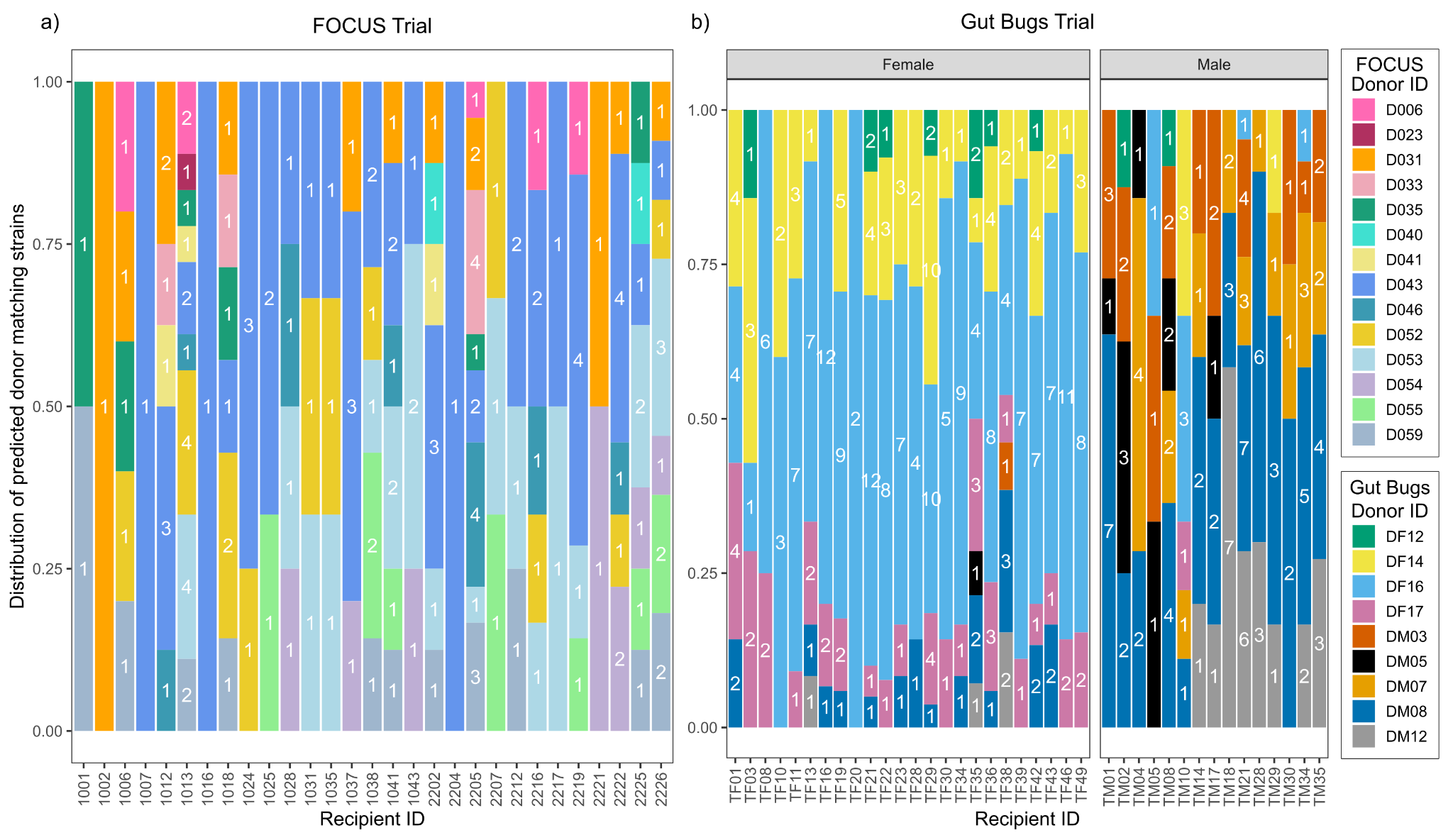


**Supplementary Figure 6. Predicted donor profile for each FMT recipient with donor-matching strains, using the optimal strain matching threshold for each trial.** Novel donor-matching strains were identified in (a) 29/32 FMT recipients of the FOCUS Trial at week 8 and (b) all 39 FMT recipients of the Gut Bugs Trial at week 6. The distribution of donors with matching strains was plotted to assign a tentative donor profile for each of these FMT recipients (FOCUS Trial range: 1-9 donors per recipient; Gut Bugs Trial range: 1-7 donors per recipient). Bars are coloured by FMT donors. Count data and bar height correspond to the number of distinct species strains matching each donor in each FMT recipient sample at the first post-intervention timepoint (FOCUS Trial: week 8, Gut Bugs Trial: week 6). FMT, faecal microbiota transplantation.


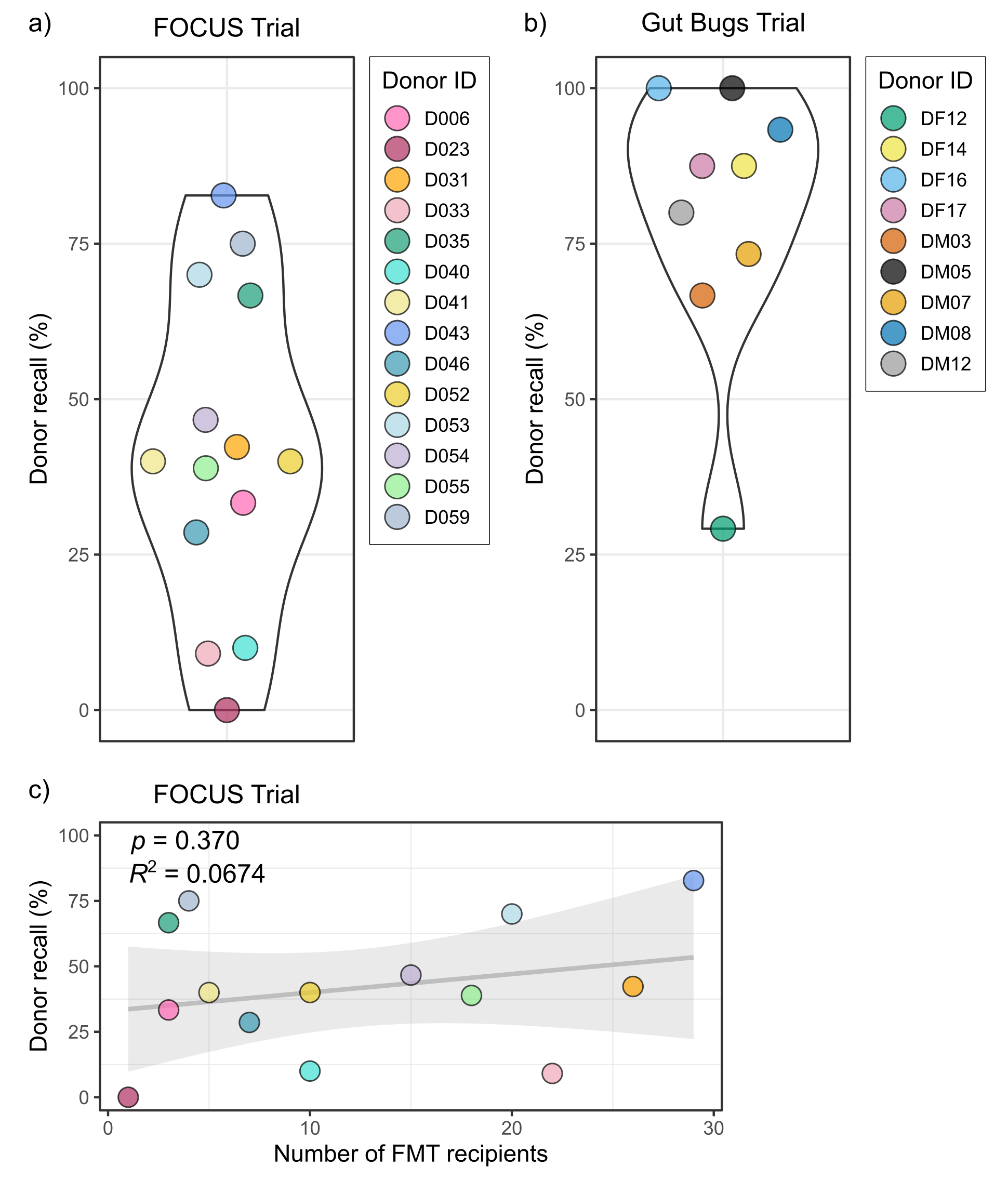


**Supplementary Figure 7. Recall was variable across donors within each FMT trial.** Donor recall was calculated as the percentage of true positive donor-recipient pairings predicted from strain engraftment analysis (TP/(TP+FN)) for each FMT donor in (a) the FOCUS Trial and (b) the Gut Bugs Trial. (c): The correlation between donor recall and donor use in the FOCUS Trial was plotted using a linear model. Points are coloured by FMT donors. The grey shaded region represents the 95% confidence interval. Degrees of freedom = 12. FMT, faecal microbiota transplantation; TP, true positive; FN, false negative.


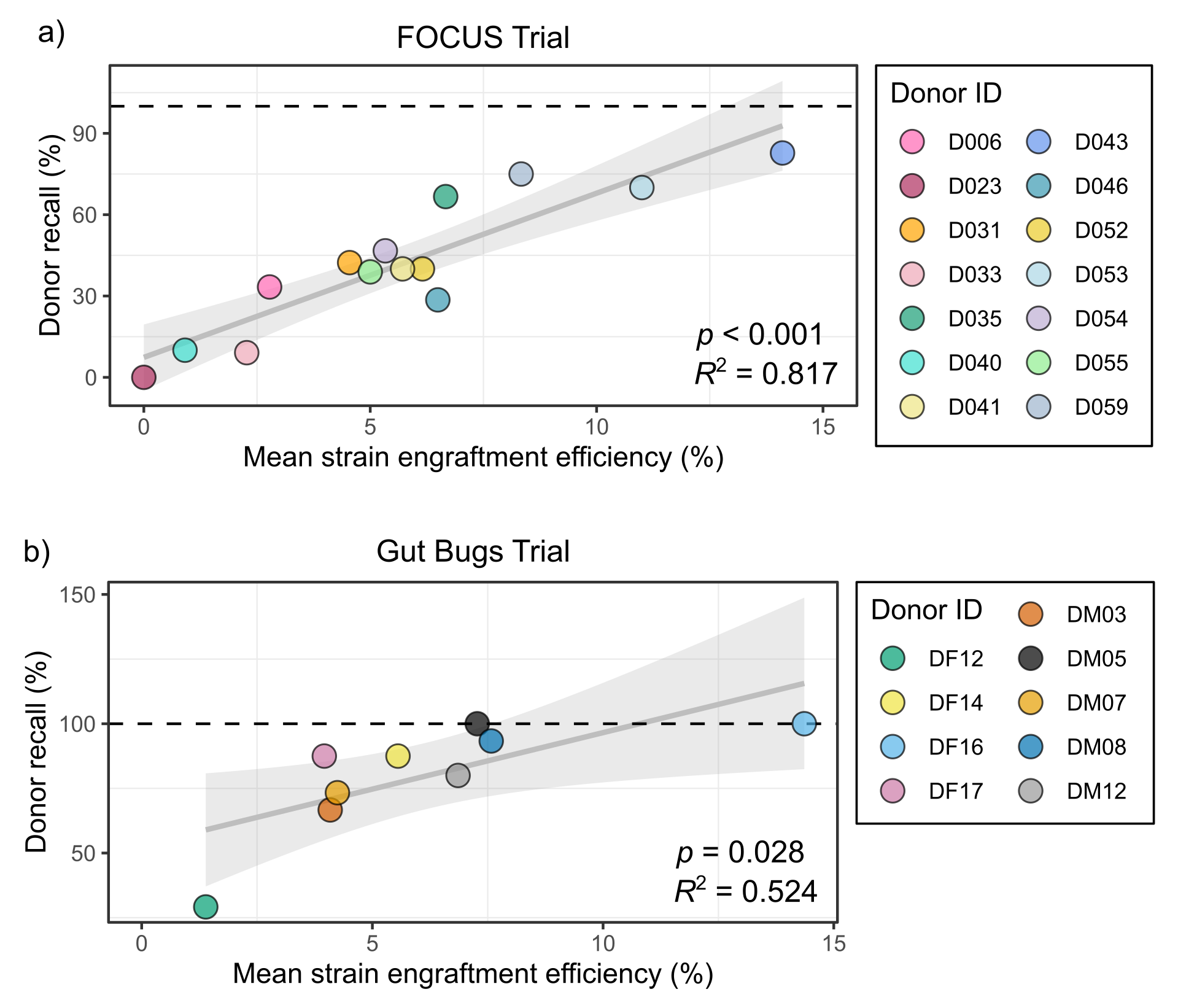


**Supplementary Figure 8. Donor recall correlated with donor engraftment efficiency.** The correlation between donor recall and engraftment efficiency was plotted using a linear model for (a) the FOCUS Trial and (b) the Gut Bugs Trial. Dashed lines represent 100% recall. Points are coloured by FMT donors. The grey shaded region represents the 95% confidence interval. FOCUS Trial degrees of freedom = 12; Gut Bugs Trial degrees of freedom = 7. FMT, faecal microbiota transplantation.

**
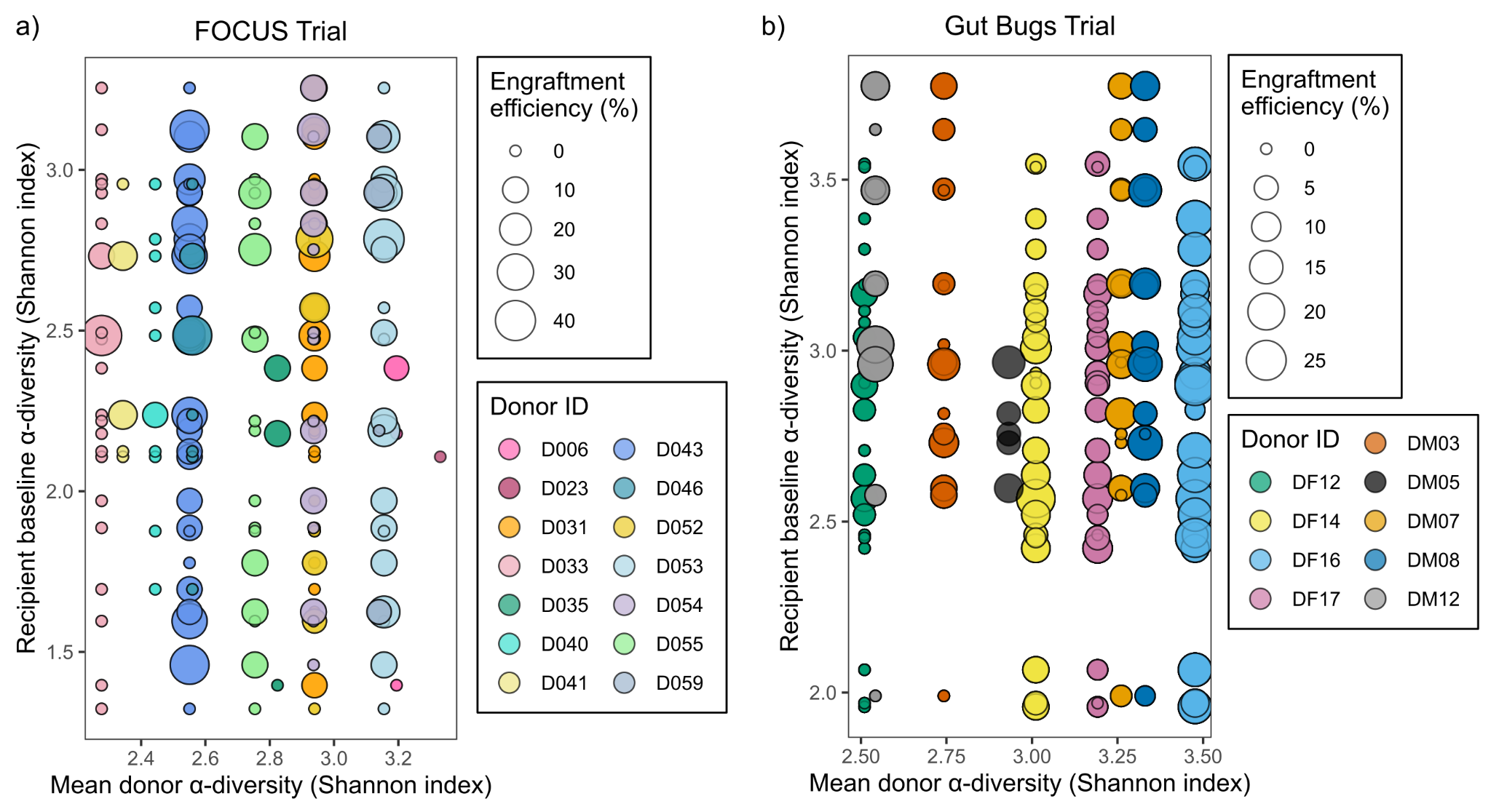
**

**Supplementary Figure 9. Donor-recipient engraftment efficiency is more consistently above zero for donors with α-diversity greater than 2.5.** The mean α-diversity of each donor’s samples was plotted against the α-diversity of their true recipients at baseline in (a) the FOCUS Trial and (b) the Gut Bugs Trial. The size of the intersecting point corresponds to the engraftment efficiency for that pairing at the first post-intervention timepoint (FOCUS Trial: week 8; Gut Bugs Trial: week 6). Points are coloured by FMT donors. FMT, faecal microbiota transplantation.


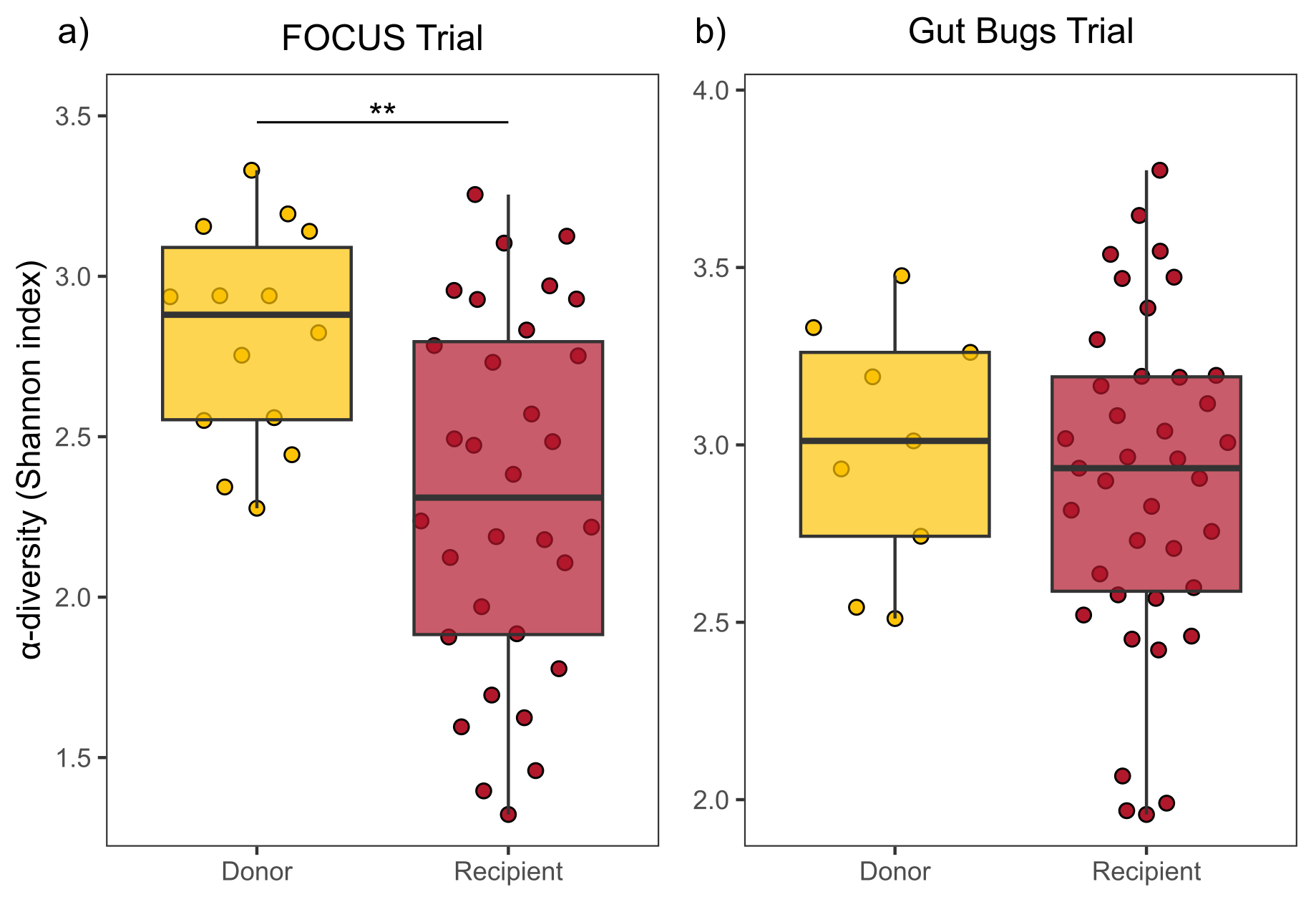


**Supplementary Figure 10. The α-diversity of recipients with UC is significantly decreased compared with healthy donors.** The mean α-diversity of each donor and α-diversity of each FMT recipient at baseline is plotted for (a) the FOCUS Trial and (b) the Gut Bugs Trial. Donor (yellow) and recipient (red) α-diversity values were compared within each trial using the Wilcoxon rank sum test. Each point represents a participant. Boxes represent the interquartile range (IQR) split by the median, with whiskers extending up to 1.5 × the IQR. UC, ulcerative colitis; FMT, faecal microbiota transplantation; ***p* < 0.01.


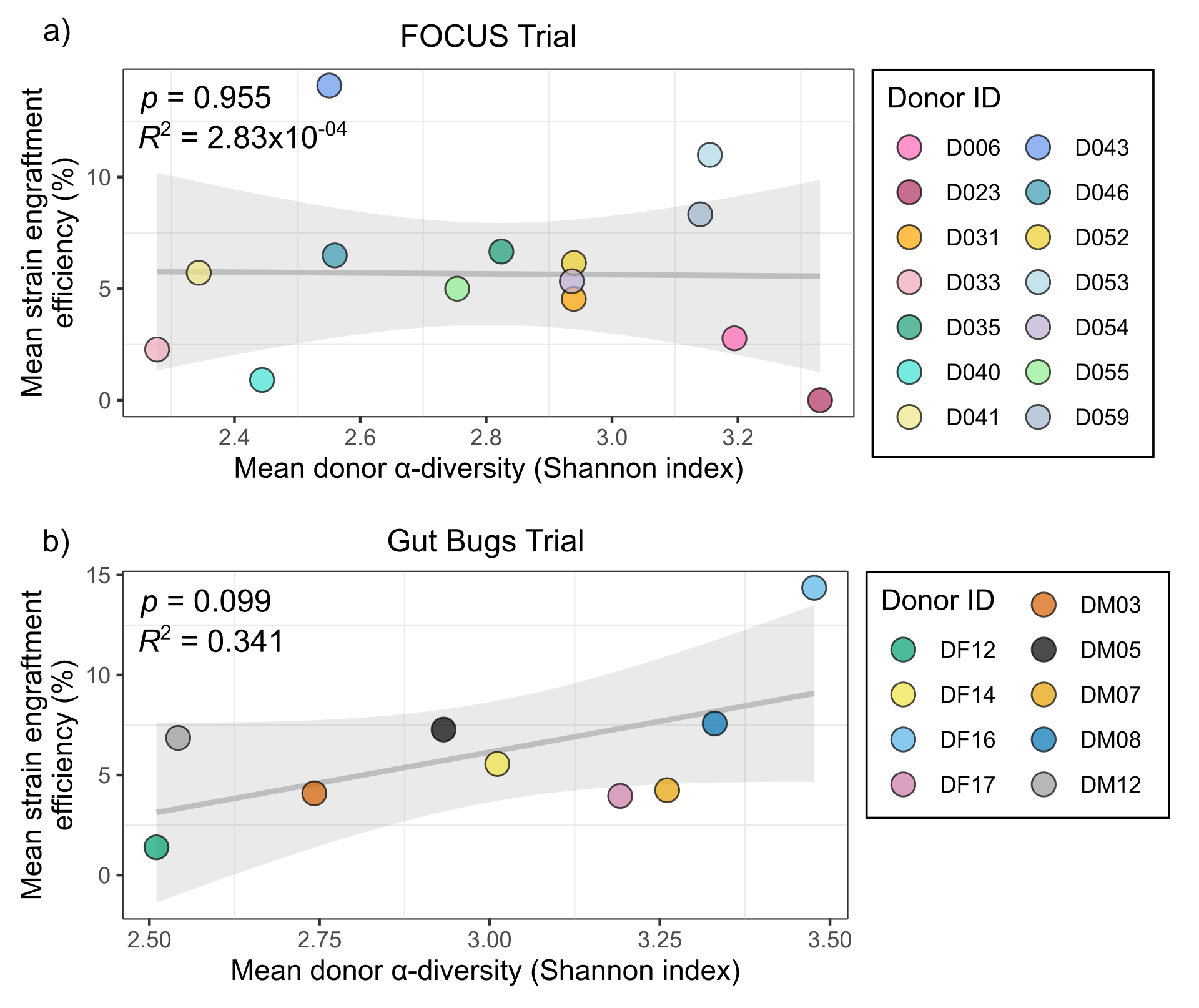


**Supplementary Figure 11. Donor engraftment efficiency was not correlated with α-diversity in either trial.** The correlation between mean donor strain engraftment efficiency and mean donor α-diversity was plotted using a linear model for (a) the FOCUS Trial and (b) the Gut Bugs Trial. Points are coloured by FMT donors. The grey shaded region represents the 95% confidence interval. FOCUS Trial degrees of freedom = 12; Gut Bugs Trial degrees of freedom = 7. FMT, faecal microbiota transplantation.


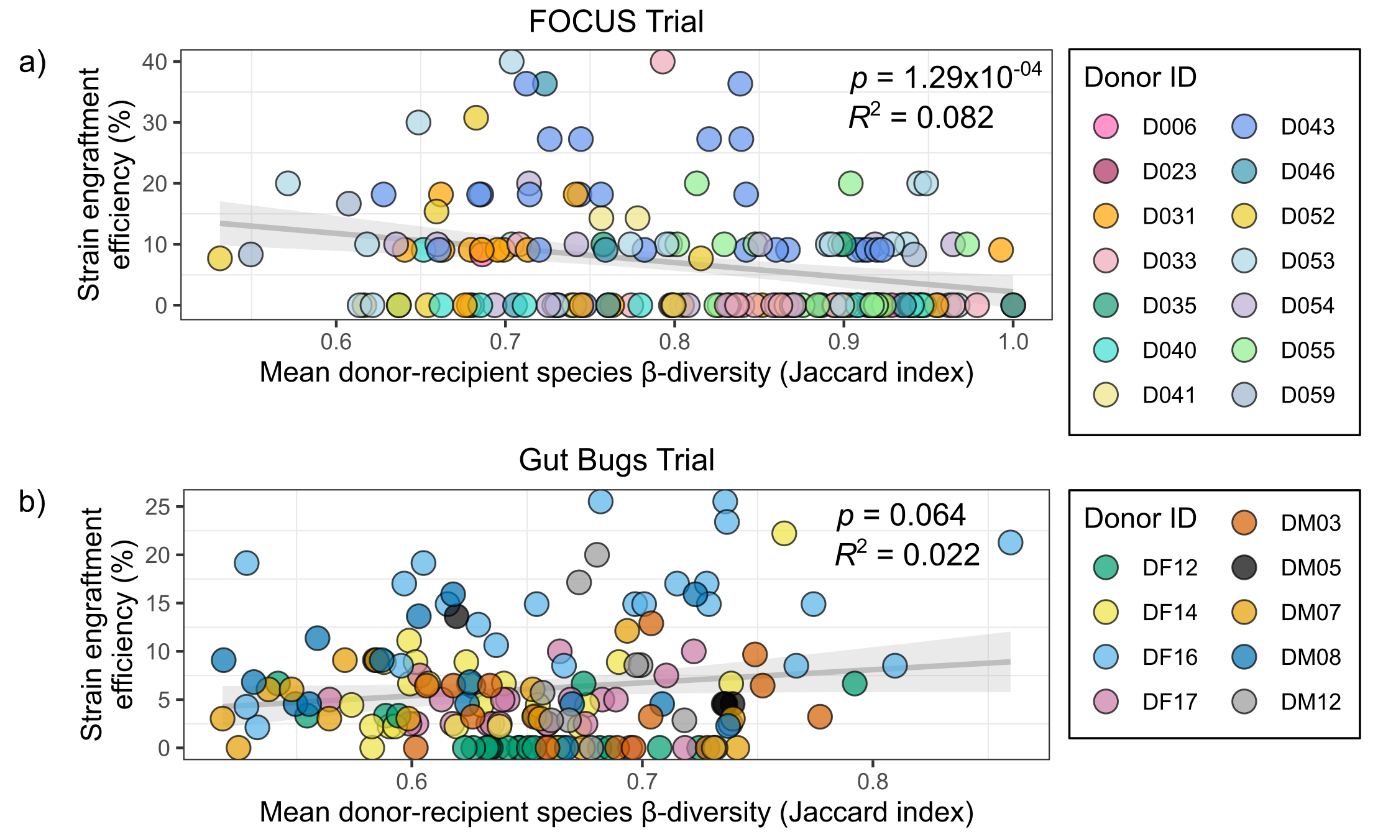


**Supplementary Figure 12.** **The correlation between donor-recipient species β-diversity (Jaccard dissimilarity index) and engraftment efficiency differed between the trials.** The correlation between strain engraftment efficiency and mean donor-recipient (baseline) species Jaccard β-diversity was plotted using a linear model for (a) the FOCUS Trial and (b) the Gut Bugs Trial. Species profiles were obtained from MetaPhlAn3. Points are coloured by FMT donors. The grey shaded region represents the 95% confidence interval. FOCUS Trial degrees of freedom = 171; Gut Bugs Trial degrees of freedom = 154. When calculating Jaccard β-diversity, the relative abundance data were first converted to presence/absence values by specifying ‘binary = TRUE’. FMT, faecal microbiota transplantation.


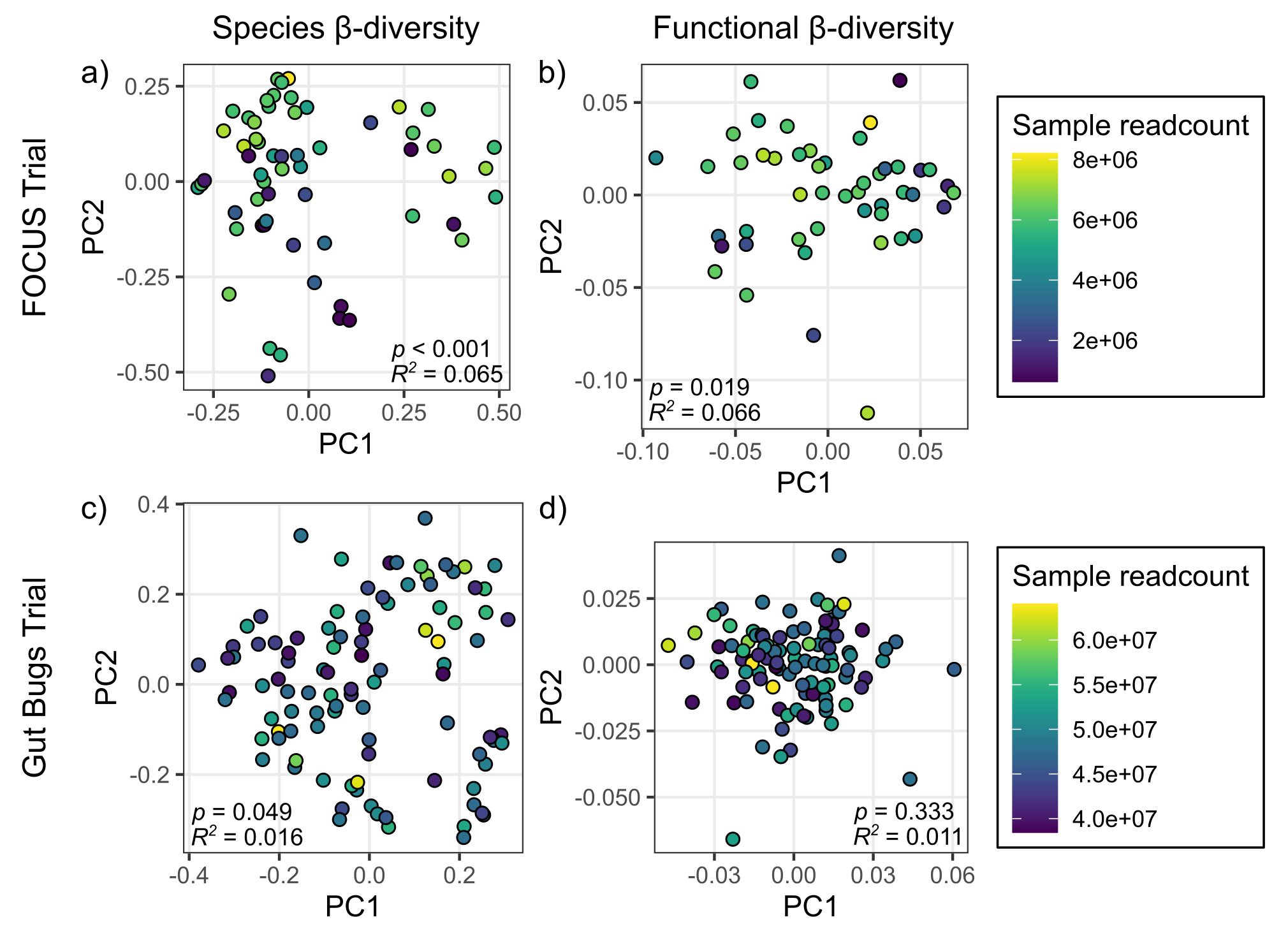


**Supplementary Figure 13. Functional and species Bray-Curtis β-diversity are significantly impacted by sample read counts.** Classical (metric) multidimensional scaling of (a) species relative abundance data and (b) COG functional category relative abundance data for all individual donor and FMT recipient baseline samples in the FOCUS Trial (*n* = 59), and (c) species relative abundance data and (d) COG functional category relative abundance data for all donor and FMT recipient baseline samples in the Gut Bugs Trial (*n* = 100), using the Bray-Curtis dissimilarity index. Species profiles were obtained from MetaPhlAn3. COG functional annotations for genes on high-quality MAGs (high-quality genes) in each sample were obtained using eggNOG. The relative abundance of each COG functional category was calculated as a proportion of the total number of high-quality genes for each sample. Relative abundance data was renormalised for each sample as some genes had multiple functional annotations. Each point represents a sample. High-quality genes with functional annotations were available for 48/59 of the FOCUS Trial sample subset and all 100 of the Gut Bugs Trial sample subset. Points are coloured by total sample read count within each trial. Coordinates are equal within each plot. PC1, principal coordinate 1; PC2, principal coordinate 2; MAG, metagenome-assembled genome; COG, clusters of orthologous groups.


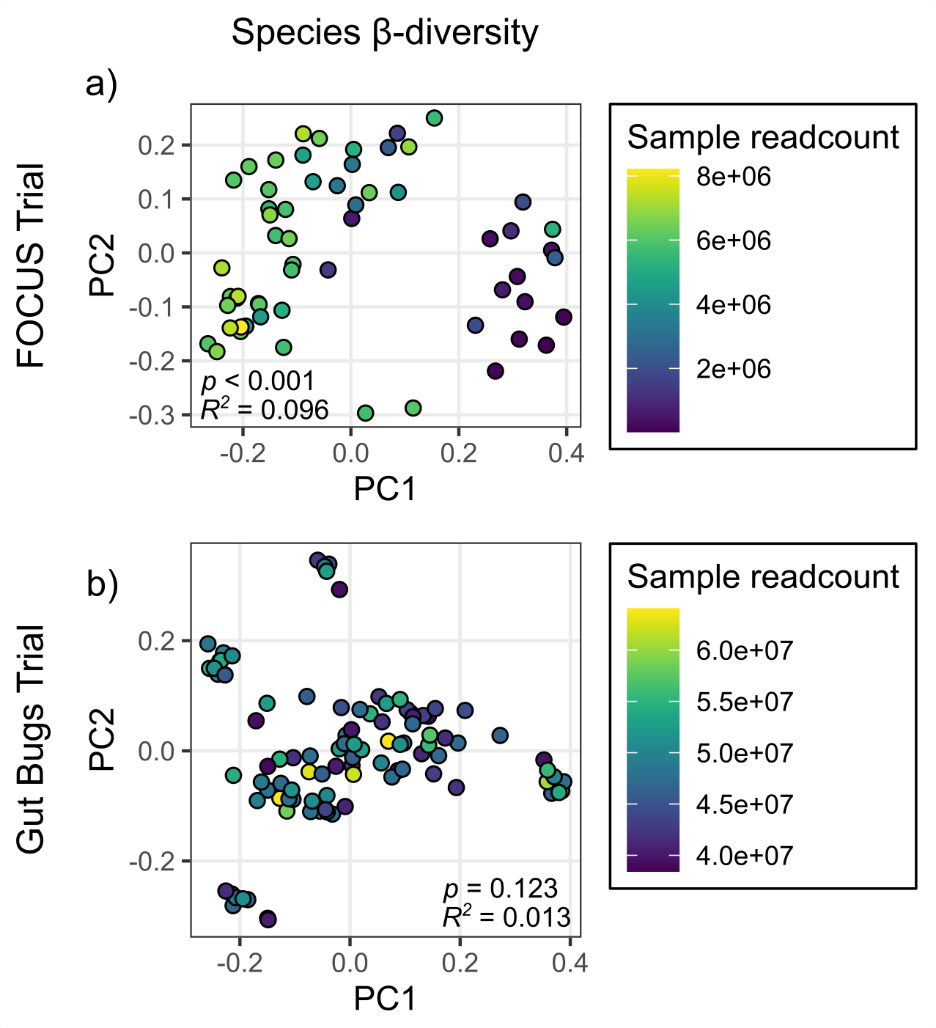


**Supplementary Figure 14. Species Jaccard β-diversity is significantly impacted by sample read counts in the FOCUS Trial.** Classical (metric) multidimensional scaling of species relative abundance data for all individual donor and FMT recipient baseline samples in (a) the FOCUS Trial (*n* = 59), and (b) all donor and FMT recipient baseline samples in the Gut Bugs Trial (*n* = 100), using the Jaccard dissimilarity index. Species profiles were obtained from MetaPhlAn3. When calculating Jaccard β-diversity, the relative abundance data were first converted to presence/absence values by specifying ‘binary = TRUE’. Each point represents a sample. Points are coloured by total sample read count within each trial. Coordinates are equal within each plot. PC1, principal coordinate 1; PC2, principal coordinate 2.
